# Biosynthesis of mycobacterial arabinogalactan: identification of a novel α(1→3) arabinofuranosyltransferase

**DOI:** 10.1111/j.1365-2958.2008.06354.x

**Published:** 2008-07-15

**Authors:** Helen L Birch, Luke J Alderwick, Apoorva Bhatt, Doris Rittmann, Karin Krumbach, Albel Singh, Yu Bai, Todd L Lowary, Lothar Eggeling, Gurdyal S Besra

**Affiliations:** 1 School of Biosciences, University of BirminghamEdgbaston, Birmingham B15 2TT, UK; 2 Institute for Biotechnology 1, Research Centre JuelichD-52425 Juelich, Germany; 3 Alberta Ingenuity Centre for Carbohydrate Science and Department of Chemistry, University of AlbertaAB, Canada T6G 2G2

## Abstract

The cell wall mycolyl-arabinogalactan–peptidoglycan complex is essential in mycobacterial species, such as *Mycobacterium tuberculosis* and is the target of several antitubercular drugs. For instance, ethambutol targets arabinogalactan biosynthesis through inhibition of the arabinofuranosyltransferases Mt-EmbA and Mt-EmbB. A bioinformatics approach identified putative integral membrane proteins, MSMEG2785 in *Mycobacterium smegmatis*, Rv2673 in *Mycobacterium tuberculosis* and NCgl1822 in *Corynebacterium glutamicum*, with 10 predicted transmembrane domains and a glycosyltransferase motif (DDX), features that are common to the GT-C superfamily of glycosyltransferases. Deletion of *M. smegmatis MSMEG2785* resulted in altered growth and glycosyl linkage analysis revealed the absence of AG α(1→3)-linked arabinofuranosyl (Ara*f*) residues. Complementation of the *M. smegmatis* deletion mutant was fully restored to a wild-type phenotype by MSMEG2785 and Rv2673, and as a result, we have now termed this previously uncharacterized open reading frame, arabinofuranosyltransferase C (*aftC*). Enzyme assays using the sugar donor β-d-arabinofuranosyl-1-monophosphoryl-decaprenol (DPA) and a newly synthesized linear α(1→5)-linked Ara_5_ neoglycolipid acceptor together with chemical identification of products formed, clearly identified AftC as a branching α(1→3) arabinofuranosyltransferase. This newly discovered glycosyltransferase sheds further light on the complexities of *Mycobacterium* cell wall biosynthesis, such as in *M. tuberculosis* and related species and represents a potential new drug target.

## Introduction

Tuberculosis (TB) affects a third of the world population and causes 1.8 million fatalities annually (Dye, 2006). The spread of TB has been facilitated in recent years due to the susceptibility of HIV-infected individuals to *Mycobacterium tuberculosis*, the aetiological agent of TB (Paolo and Nosanchuk, 2004). The problem has also been compounded by the emergence of multidrug-resistant TB (MDR-TB) (Kaye and Frieden, 1996) and extensively drug-resistant (XDR)-TB strains (Shah *et al*., 2007). *M. tuberculosis* and other mycobacteria have a distinct cell wall which has a lipid-rich outer layer that is highly impermeable (Minnikin, 1982). One of the major components of this outer envelope are mycolic acids, long-chain α-alkyl, β-hydroxy fatty acids that are essential for bacterial survival (Vilcheze *et al*., 2000; Portevin *et al*., 2004; Bhatt *et al*., 2005; Parish *et al*., 2007). These are found either esterified to the non-reducing termini of arabinogalactan (AG), or are present as trehalose esters, such as trehalose dimycolate (TDM) (Brennan and Nikaido, 1995; Dover *et al*., 2004).

A common feature of members of the *Corynebacterianeae* is that they all possess this unusual cell wall architecture (McNeil *et al*., 1990; 1991; Besra *et al*., 1995). Apart from mycolic acids, the cell wall is dominated by a second macromolecule, an essential heteropolysaccharide termed AG, which is linked to both mycolic acids and peptidoglycan, forming the mycolyl-arabinogalactan–peptidoglycan (mAGP) complex (Daffé *et al*., 1990; McNeil *et al*., 1990; 1991; Besra *et al*., 1995). The formation of the arabinan domain (α1→5, α1→3 and β1→2 glycosyl linkages) of AG results from the subsequent addition of arabinofuranose (Ara*f*) residues by a set of unique arabinofuranosyltransferases including, the Emb proteins of which three paralogues exist in *Mycobacterium avium* (Belanger *et al*., 1996) and *M. tuberculosis* (Telenti *et al*., 1997), AftA (Alderwick *et al*., 2006a) and AftB (Seidel *et al*., 2007a). The lipid linked sugar donor β-d-arabinofuranosyl-1-monophosphoryldecaprenol (DPA; Wolucka *et al*., 1994; Lee *et al*., 1995; 1997) serves as the substrate molecule for these complex membrane-bound glycosyltransferases.

**Fig. 1:**
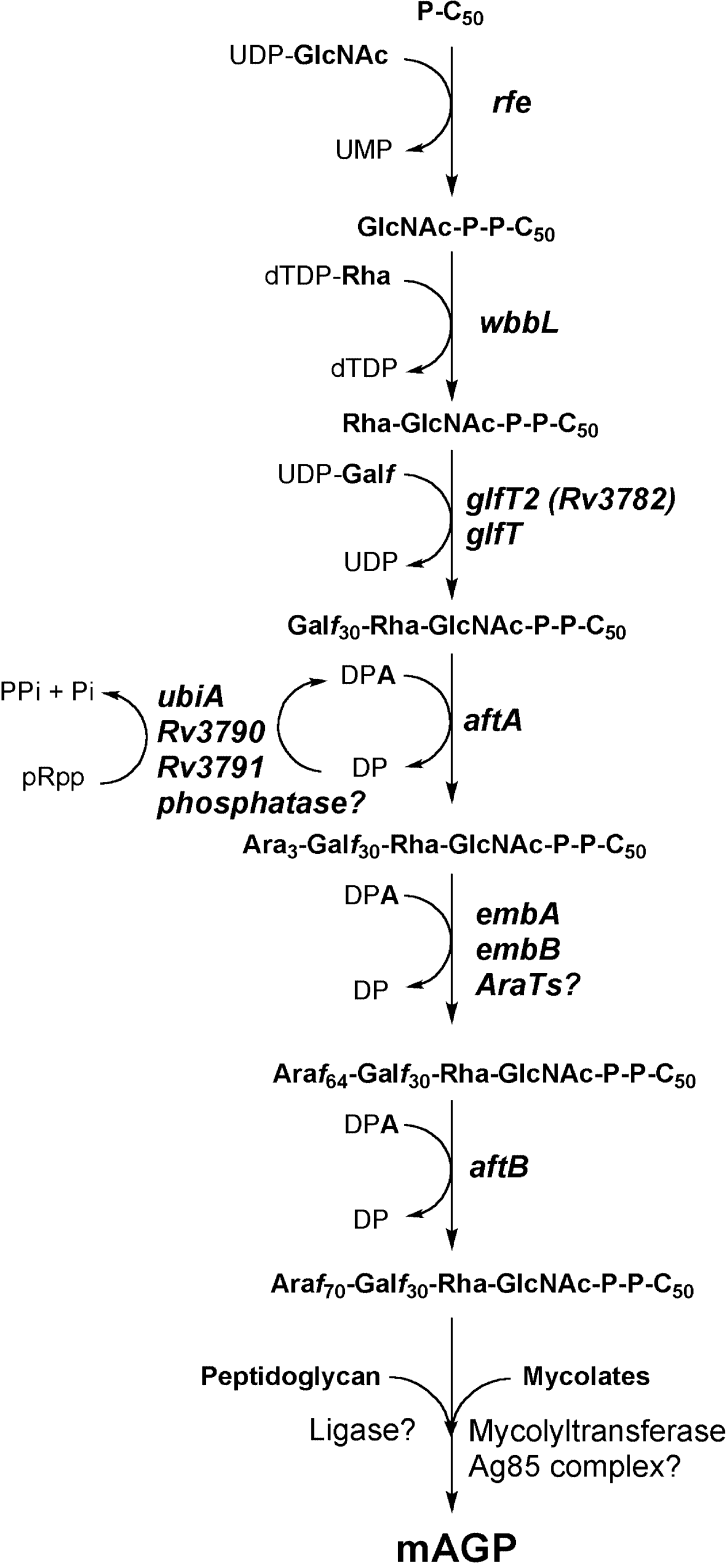
Biosynthetic pathway leading to arabinan formation in *M. tuberculosis* AG.

The antituberculosis drug ethambutol (EMB) was shown to specifically inhibit AG biosynthesis (Takayama and Kilburn, 1989). The precise molecular target of EMB occupies the *embCAB* locus in *M. tuberculosis* (Telenti *et al*., 1997). To further define the role of EmbCAB proteins in cell wall arabinan biosynthesis, *embA*, *embB* and *embC* were individually inactivated in *Mycobacterium smegmatis* (Escuyer *et al*., 2001; Zhang *et al*., 2003). All three mutants were viable; however, the non-reducing terminal Ara_6_ motif which is the template for mycolylation in AG (McNeil *et al*., 1991) was altered in both the *M. smegmatis* (Ms)-*embA* and Ms-*embB* mutants (Escuyer *et al*., 2001), while *Ms-embC* was shown to be involved in the formation of the arabinan domains of lipoarabinomannan (LAM; Zhang *et al*., 2003). Attempts to obtain deletion mutants of *embA* (Amin *et al*., 2008) and *embB* in *M. tuberculosis* and *embAB* in *M. smegmatis* have proved unsuccessful (G.S. Besra, unpubl. results). In contrast, deletion of the single *C. glutamicum* (Cg)*-emb* orthologue and chemical analysis of the cell wall revealed a novel truncated AG structure possessing only terminal (t)-Ara*f* residues with a corresponding loss of cell wall-bound mycolic acids (Alderwick *et al*., 2005). The presence of a novel enzyme responsible for ‘priming’ the galactan domain for further elaboration by Emb proteins led to the identification of AftA (Alderwick *et al*., 2006a). Recently, a retaining GT-C enzyme was identified, now termed AftB, which is responsible for the attachment of terminal β(1→2) Ara*f* residues, and marks the ‘end-point’ for AG arabinan biosynthesis (Fig. 1) before decoration with mycolic acids (Seidel *et al*., 2007a).

It is clear that additional arabinofuranosyltransferases involved in AG and LAM biosynthesis still remain to be identified. Indeed, Liu and Mushegian (2003) identified 15 members of the GT-C superfamily residing in *M. tuberculosis*, representing candidates involved in the biosynthesis of cell wall-related glycans and lipoglycans (Liu and Mushegian, 2003). We have continued our earlier studies (Alderwick *et al*., 2006a,b; Seidel *et al*., 2007a) to identify genes required for the biosynthesis of the core structural elements of the mAGP complex by studying mutants of *M. smegmatis* and the orthologous genes and enzymes of *M. tuberculosis and Corynebacterium glutamicum*. Herein, we present MSMEG2785, Rv2673 and NCgl1822 as a new distinct arabinofuranosyltransferase of the GT-C superfamily, which is responsible for the transfer of Ara*f* residues from DPA to the arabinan domain to form α(1→3)-linked Ara*f* residues, which result in the branched arabinan domain distal to the non-reducing terminal Ara_6_ motif characteristic of mycobacterial AG.

## Results

### Genome comparison of the Rv2673 locus

**Fig. 2:**
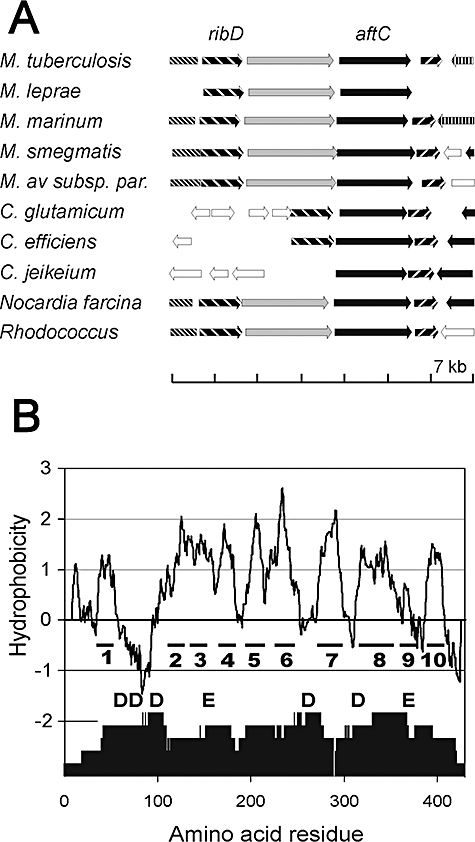
Comparison of the *aftC* locus within the *Corynebacterianeae*. A. The locus in the bacteria analysed consists of *aftC* which in *M. tuberculosis* has the locus tag Rv2673 and in *C. glutamicum* NCgl1822. The genomic region displayed encompasses 7 kb, and orthologous genes are highlighted accordingly. *M. marinum*, *Mycobacterium marinum*; *M. av* ssp. *par.*, *Mycobacterium avium* ssp. *paratuberculosis*; *C. efficiens*, *Corynebacterium efficiens*; *C. jeikeium*, *Corynebacterium jeikeium; Nocardia farcina*, *Nocardia farcina* IFM 10 152; *Rhodococcus*, *Rhodococcus* sp. strain RHA1. B. AftC is a hydrophobic protein predicted to span the membrane 10 times and the transmembrane helices are numbered accordingly. The lower part of the figure shows the degree of conservation of the orthologues given in A as analysed by the DIALIGN method (Brudno *et al*., 2003). Also shown is the approximate position of the fully conserved aspartyl (D) and glutamyl (E) residues.

The arabinofuranosyltransferases EmbA, EmbB and EmbC are vital for *M. tuberculosis* and represent a target for the established drug EMB (Mikusova *et al*., 1995; Belanger *et al*., 1996; Telenti *et al*., 1997). Structural considerations of these proteins and a search for new drug targets resolved that more than 16 related proteins are present in *M. tuberculosis,* possibly also acting as glycosyltransferases (Liu and Mushegian, 2003). In our systematic analysis of GT-C glycosyltransferases, focusing on those present in *M. tuberculosis* and *C. glutamicum*, we have previously identified the arabinofuranosyltransferases AftA (Alderwick *et al*., 2006a) and AftB (Seidel *et al*., 2007a), as well as several α-mannosyltransferases (Mishra *et al*., 2007; 2008). Each of these glycosyltransferases plays a specific yet decisive role in cell wall biosynthesis and assembly. *In silico* analysis of one of the putative glycosyltransferases of *M. tuberculosis*, Rv2673, highlighted that orthologues are present in a range of species belonging to the suborder *Corynebacterianeae*, including the families *Mycobacteriaceae*, *Corynebacteriacea* and *Nocardiaceae* (Fig. 2A). Furthermore, the organization of the gene locus is largely retained. The adjacent genes are largely of unknown function. *RibD* encodes a bifunctional deaminase–reductase domain, followed by a gene product containing a hydrolase domain, which is however, absent in *Corynebacterium*, and downstream of Rv2673 a gene of unknown function is present. The wide distribution of *Rv2673*, its syntenic organization, and the fact that it is retained even in *M. leprae*, strongly indicates a fundamental function of its product. According to our experimental analysis (see below) we annotated this gene arabinofuranosyltransferase C (*aftC*).

AftC of *M. tuberculosis* is 433 amino acid residues long. It is a hydrophobic protein and is predicted to possess 10 transmembrane-spanning segments (Fig. 2B). However, in contrast to AftA, AftB or EmbC, it is characterized by the absence of a periplasmic carboxyterminal extension. The amino acid sequence among the *Corynebacterianeae* is very well conserved, and there are 43% identical residues shared by the *M. tuberculosis* and *C. glutamicum* proteins. The degree of conservation is particularly high in the loop regions, for instance between helixes 1 and 2, 3 and 4, or 6 and 7 (Fig. 2B). The fully conserved aspartyl (D) and glutamyl (E) residues, which we propose to be involved in catalysis or substrate binding, are located in the first extended loop region (Liu and Mushegian, 2003), as we have demonstrated for similarly located aspartyl (D) residues of Cg-Emb and AftB (Seidel *et al*., 2007a,b). Interestingly, the long transmembrane helix 8 is well conserved and it is within this region that there is a strong identity to a membrane protein of *Vibrio parahaemolyticus* (CpsG). Furthermore, this gene is located in a gene cluster involved in the biosynthesis of a capsular polysaccharide within this pathogen (Guvener and McCarter, 2003).

### Construction and growth of mutants

**Fig. 3:**
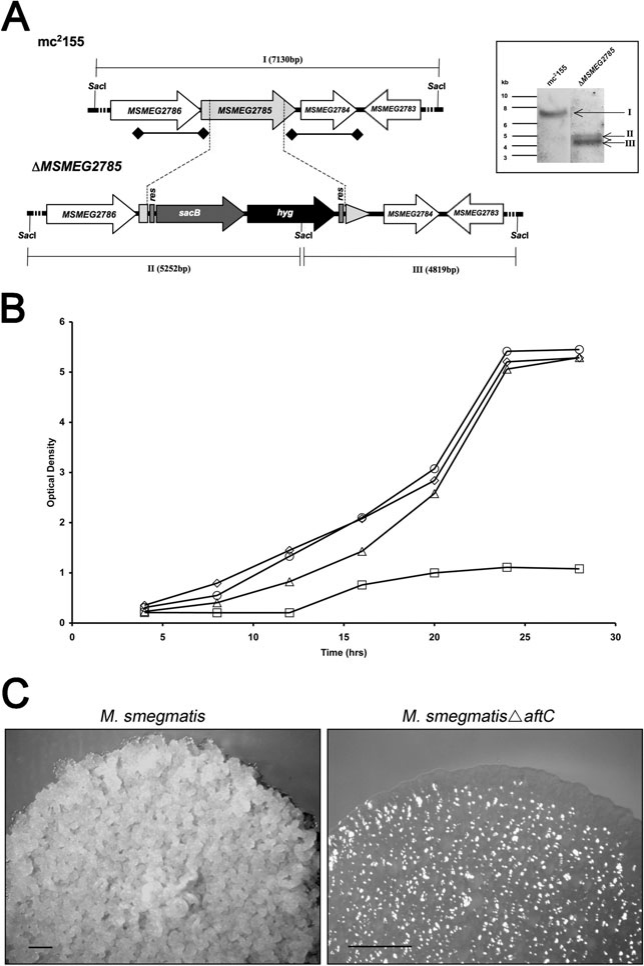
Generation of a *MSMEG2785* null mutant. A. A map of the *MSMEG2785* region in the parental *M. smegmatis* strain and its corresponding region in the Δ*MSMEG2785* mutant. *res*, γδ resolvase site; *hyg*, hygromycin resistance gene from *Streptomyces hygroscopicus*; *sacB*, sucrose counter-selectable gene from *Bacillus subtilis*. Digoxigenin-labelled probes were derived from ∼1 kb upstream and downstream flanking sequences that were used to construct the knockout plasmid, and are indicated by thick lines with square ends. SacI-digested bands expected in a Southern blot are indicated in roman numerals with sizes in brackets. The inset shows the Southern blot of SacI-digested genomic DNA from the two strains with expected bands indicated by arrows. B. Growth of wild-type *M. smegmatis* (◊), *M. smegmatis***Δ***aftC* (□), *M. smegmatis***Δ***aftC* pMV261-Ms-*aftC* (▵) and *M. smegmatis***Δ***aftC* pMV261-Mt-*aftC* (○) in TSB medium. C. Colony morphology of wild-type *M. smegmatis* and *M. smegmatis*Δ*aftC* on TSB-agar plates. Black bar represents 1 mm.

In order to delete *aftC* and study for possible consequences we generated a null mutant of *M. smegmatis* mc^2^155 *MSMEG2785* (orthologue of *Rv2673*) using specialized transduction (Fig. 3A). In contrast to our *C. glutamicum* studies (see below) growth of *M. smegmatis*Δ*aftC* in comparison to *M. smegmatis* was poor in liquid medium (Fig. 3B) and sensitive to the addition of Tween-80 on agar plates (> 0.005%). Complementation of *M. smegmatis*Δ*aftC* with either pMV261-Ms-*aftC* or pMV261-*M. tuberculosis* (Mt)-*aftC* restored the mutant to a wild-type phenotype (Fig. 3B). On solid media *M. smegmatis*Δ*aftC* had a smooth and glossy appearance in comparison to the typical crenulated colony morphology found for wild-type *M. smegmatis* (Fig. 3C) and failed to stain as ‘acid-fast’ positive (data not shown). In addition, susceptibility of *M. smegmatis*Δ*aftC* to EMB and the hydrophobic antibiotics rifampicin and chloramphenicol was enhanced (minimal inhibitory concentration of 2, 100 and 10 μg ml^−1^ for wild-type *M. smegmatis* in comparison to 0.4, 4 and 5 μg ml^−1^ for *M. smegmatis*Δ*aftC* respectively), indicating increased permeability and that *M. smegmatis*Δ*aftC* had an altered cell wall. To study the function of the corynebacterial AftC the non-replicative plasmid pK19mobsacBΔ*aftC* was constructed. This was used to transform *C. glutamicum* to kanamycin resistance, indicating integration in its chromosome (Fig. S1A). Loss of vector was obtained by selection for sucrose-resistance yielding clones with *aftC* deleted. A PCR analysis with primer pairs P5 and P6 resulted in the expected fragment of 2160 bp for the wild-type and of 1065 bp for the deletion mutant, which was termed *C. glutamicum*Δ*aftC*. Colonies of this mutant were more erose compared with the usual glossy appearance of the wild-type colony (data not shown). In contrast to *M. smegmatis*Δ*aftC* the growth of the *C. glutamicum*Δ*aftC* mutant on the salt medium CGXII possessed only a slightly reduced growth rate of 0.32 h^−1^, whereas, that of the wild-type *C. glutamicum* was 0.39^−1^ h (Fig. S1B).

### mAGP analyses from *M. smegmatis*, *M. smegmatisΔaftC*, *M. smegmatisΔaftC* pMV261-Ms-*aftC*, *M. smegmatisΔaftC* pMV261-Mt-*aftC*, *C. glutamicum* and *C. glutamicumΔaftC*

**Fig. 4:**
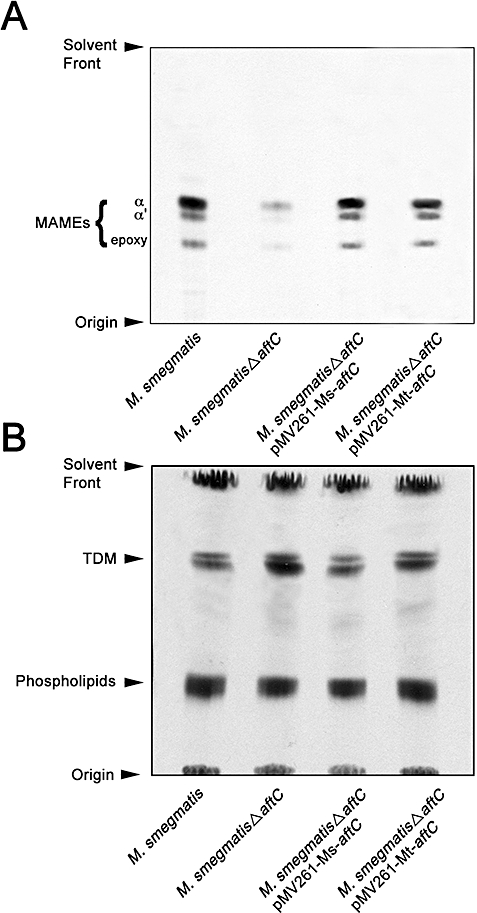
Analysis of cell wall-associated lipids and bound MAMEs from *M. smegmatis*, *M. smegmatis*Δ*aftC*, *M. smegmatis*Δ*aftC* pMV261-Ms-*aftC* and *M. smegmatis*Δ*aftC* pMV261-Mt-aftC. A. Analysis of cell wall-bound MAMEs from *M. smegmatis*, *M. smegmatis*Δ*aftC*, *M. smegmatis*Δ*aftC* pMV261-Ms-*aftC* and *M. smegmatis*Δ*aftC* pMV261-Mt-*aftC*. The bound mycolic acids from an equivalent amount of freeze-dried cells (100 mg), which were initially de-lipidated using two consecutive extractions of CHCl_3_/CH_3_OH/H_2_O (10/10/3; v/v/v) at 50°C for 4 h, were released by the addition of tetra-butylammonium hydroxide at 100°C overnight, and methylated as described in the *Experimental procedures*. An equivalent aliquot from each strain was subjected to TLC using silica gel plates (5735 silica gel 60F254, Merck), and developed in petroleum ether/acetone (95:5, v/v) and charred to reveal MAMEs and compared with known standards (Gande *et al*., 2004). B. Quantitative analysis of extractable [^14^C]-lipids from *M. smegmatis*, *M. smegmatis*Δ*aftC*, *M. smegmatis*Δ*aftC* pMV261-Ms-*aftC* and *M. smegmatis*Δ*aftC* pMV261-Mt-*aftC*. Lipids were extracted from cells by a series of organic washes as described in *Experimental procedures* (Seidel *et al*., 2007a). An equivalent aliquot (50 000 cpm) from each strain was subjected to TLC using silica gel plates (5725 silica gel 60F254, Merck) developed in CHCl_3_/CH_3_OH/NH_4_OH (80:20:2, v/v/v) and quantified using phosphorimaging and compared with known standards (Mikusova *et al*., 1995) after exposure to Kodak X-Omat film for 24 h.

To study the function of mycobacterial *aftC* deletion, defatted cells were analysed qualitatively for AG esterified mycolic acids and cell wall-associated lipids from an equivalent starting amount of biomass for each strain due to differences in growth rate (Fig. 3B). As expected, *M. smegmatis* exhibited a typical profile of cell wall-bound α, α′ and epoxy-mycolic acid methyl esters (MAMEs), whereas, these products were drastically reduced in *M. smegmatis*Δ*aftC* (Fig. 4A). In addition, complementation of *M. smegmatis*Δ*aftC* with either pMV261-Ms*-aftC* or pMV261-Mt*-aftC* (Fig. 4A), led to the restoration of normal ‘levels’ of cell wall-bound mycolic acids. Analysis of cell wall-associated lipids in several independent experiments highlighted an apparent increase in TDM for the *aftC* deletion mutant. This was confirmed quantitatively through [^14^C]acetate labelling of cultures and equal loading of radioactivity of extractable free lipids from *M. smegmatis*, *M. smegmatis*Δ*aftC* and the complemented *M. smegmatis*Δ*aftC* strain using plasmids pMV261-Ms-*aftC* and pMV261-Mt-*aftC* (Fig. 4B). Typically, wild-type *M. smegmatis* synthesized 5250 cpm, whereas *M. smegmatis*Δ*aftC* afforded 14 676 cpm of TDM after equivalent loading of radioactivity and quantitative analysis by phosphorimaging. Complementation of *M. smegmatis*Δ*aftC* with either pMV261-Ms-*aftC* or pMV261-Mt-*aftC* restored the phenotype of the deletion mutant back to that of wild-type *M. smegmatis* (Fig. 4B). These results demonstrated that Ms-*aftC* and Mt-*aftC* are involved in a key aspect of arabinan biosynthesis, whereby deletion substantially perturbs tethering of mycolic acids to AG, which results in an increase in TDM production.

**Fig. 5:**
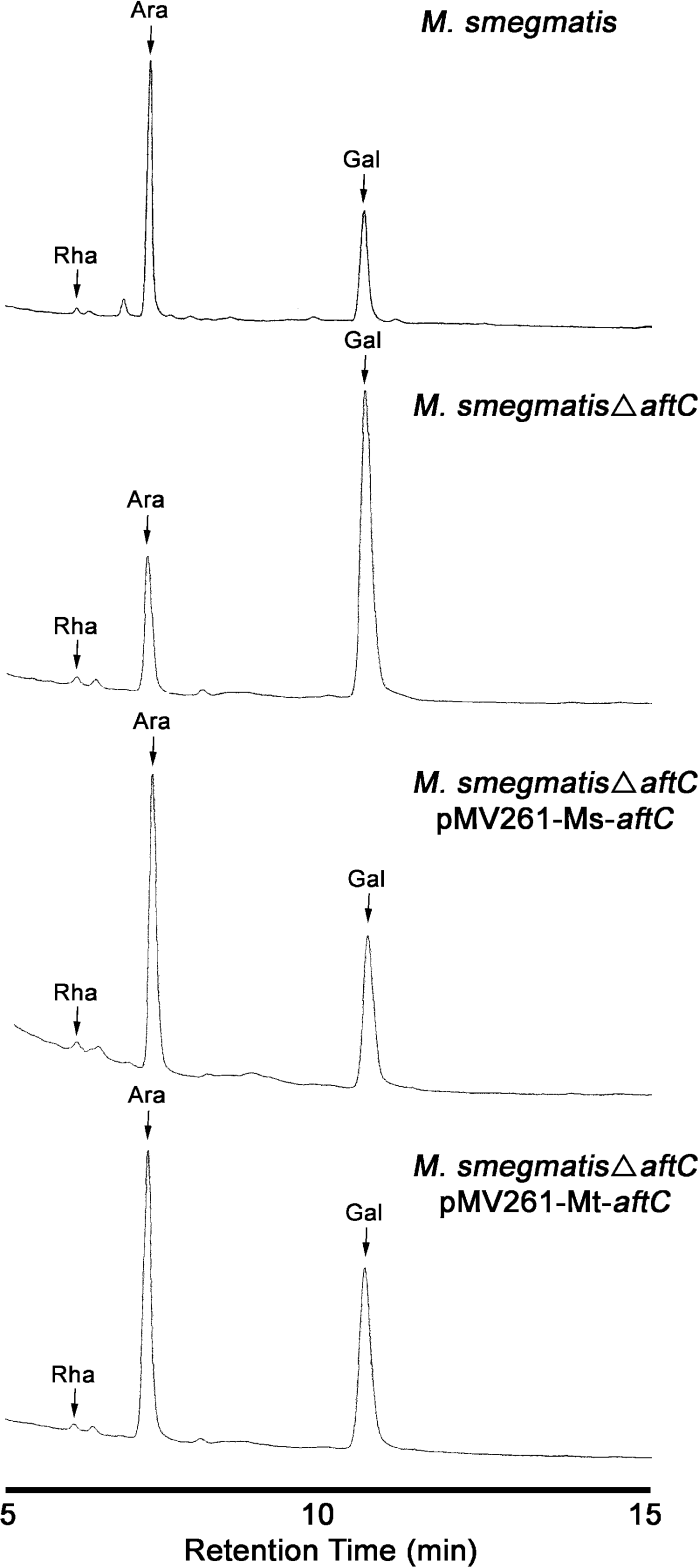
GC analysis of cell walls of *M. smegmatis*, *M. smegmatis*Δ*aftC*, *M. smegmatis*Δ*aftC* pMV261-Ms-*aftC* and *M. smegmatis*Δ*aftC* pMV261-Mt-*aftC*. Samples of purified cell walls were hydrolysed with 2 M TFA, reduced, per-*O*-acetylated and analysed as described under *Experimental procedures* (Besra *et al*., 1995; Alderwick *et al*., 2005).

**Fig. 6:**
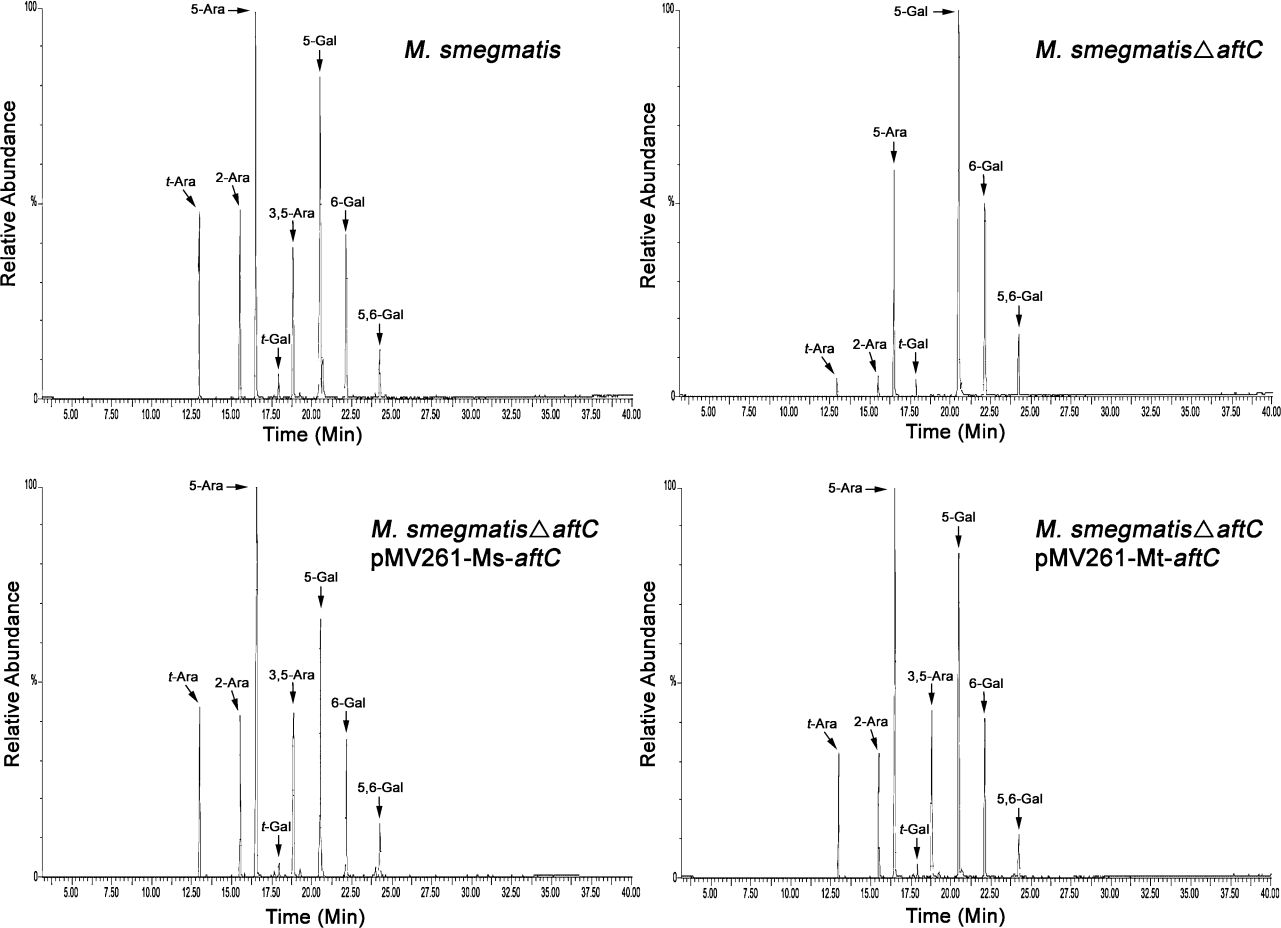
GC/MS analysis of cell walls of *M. smegmatis*, *M. smegmatis*Δ*aftC*, *M. smegmatis*Δ*aftC* pMV261-Ms-*aftC* and *M. smegmatis*Δ*aftC* pMV261-Mt-*aftC*. Samples of per-*O*-methylated cell walls were hydrolysed with 2 M TFA, reduced, per-*O*-acetylated and analysed as described under *Experimental procedures* (Besra *et al*., 1995; Alderwick *et al*., 2005).

The cell wall core (mAGP) was prepared from *M. smegmatis* and *M. smegmatis*Δ*aftC* as described (Daffé *et al*., 1990; Besra *et al*., 1995; Alderwick *et al*., 2005) and the ratio of Ara to Gal in mAGP determined by gas chromatography (GC) analysis of alditol acetates (Daffé *et al*., 1990; Besra *et al*., 1995; Escuyer *et al*., 2001; Alderwick *et al*., 2005) (Fig. 5). The glycosyl composition was calculated based on a single rhamnosyl (Rha) residue per AG chain (McNeil *et al*., 1990). The glycosyl compositional analysis revealed a relative molar ratio of Rha : Ara : Gal of 1:71:31 and an Ara : Gal ratio of 2.3:1, which is in accord with previous data (Escuyer *et al*., 2001). The *M. smegmatis*Δ*aftC* mutant yielded AG with a significant reduction in Ara content concomitant with a relative increase in the amount of Gal. The *M. smegmatis*Δ*aftC* yielded an AG with an Rha : Ara : Gal ratio of 1:22:56 and an Ara : Gal ratio of 0.4:1. Complementation of *M. smegmatis*Δ*aftC* with either pMV261-Ms*-aftC* or pMV261-Mt*-aftC*, restored the Rha : Ara : Gal ratio to that of wild-type *M. smegmatis*. Gas chromatography mass spectrometry (GC/MS) analysis of per-*O*-methylated alditol acetate derivatives prepared from *M. smegmatis* and *M. smegmatis*Δ*aftC* indicated the complete absence of 3,5-Ara*f* branching residues and a significant reduction in *t*-Ara*f*, 2-Ara*f* and 5-Ara*f*-linkages (Fig. 6). Complementation of *M. smegmatis*Δ*aftC* with either plasmid encoding Ms-*aftC* or Mt-*aftC* restored the glycosyl linkage profile to that of wild-type *M. smegmatis* (Fig. 6). These results demonstrate that MSMEG2785 and Rv2673 are functionally equivalent and are involved in the synthesis of 3,5-Ara*f* branching residues. Interestingly, LAM preparations from *M. smegmatis*Δ*aftC* were truncated in size on SDS-PAGE analysis to ‘full-size’ LAM from wild-type *M. smegmatis*. Further purification and detailed chemical analyses of LAM from the *aftC* mutant strain are currently being undertaken and will be reported separately (H.L. Birch, unpubl. results).

In contrast to the mycolic acid studies performed with the mycobacterial *aftC* deletion mutant, *C. glutamicum*Δ*aftC* cells were analysed quantitatively for AG esterified corynemycolic acids due to similar growth rates between strains (Fig. S1B). Wild-type *C. glutamicum* exhibited the known profile of corynomycolic acid methyl esters (CMAMEs, 35 345 cpm; Fig. S2), whereas, cell wall-bound CMAMEs were significantly reduced in *C. glutamicum*Δ*aftC* (8023 cpm). The above data were reassuring as the qualitative (*M. smegmatis*Δ*aftC*) and quantitative (*C. glutamicum*Δ*aft*C) analyses were comparable in terms of a reduction in cell wall-bound mycolic acids (Fig. 4A and Fig. S2). Importantly, these results have also shown that Cg-*aftC* is involved in a key aspect of arabinan biosynthesis, whereby deletion perturbs tethering of corynomycolic acids to AG. The GC/MS profiles of per-*O*-methylated alditol acetate derivatives of *C. glutamicum* and *C. glutamicum*Δ*aftC* are shown in Fig. S3 with *C. glutamicum*Δ*aftC* also clearly devoid of 3,5-Ara*f* branching residues.

### *In vitro* arabinofuranosyltransferase activity with extracts of *M. smegmatis*, *M. smegmatisΔaftC* and complemented strains

Initial attempts to develop an *in vitro* assay using either purified recombinant expressed AftC or *E. coli* membranes expressing *aftC*, have thus far proved unsuccessful, probably due to the hydrophobic nature of the protein. In an alternative approach, we assessed the capacity of membrane preparations from *M. smegmatis*, *M. smegmatis*Δ*aftC* and *M. smegmatis*Δ*aftC* complemented with pMV261-Mt-*aftC* to catalyse arabinofuranosyltransferase activity in the presence of exogenous synthetic acceptors (Lee *et al*., 1997; Seidel *et al*., 2007a).

**Fig. 7:**
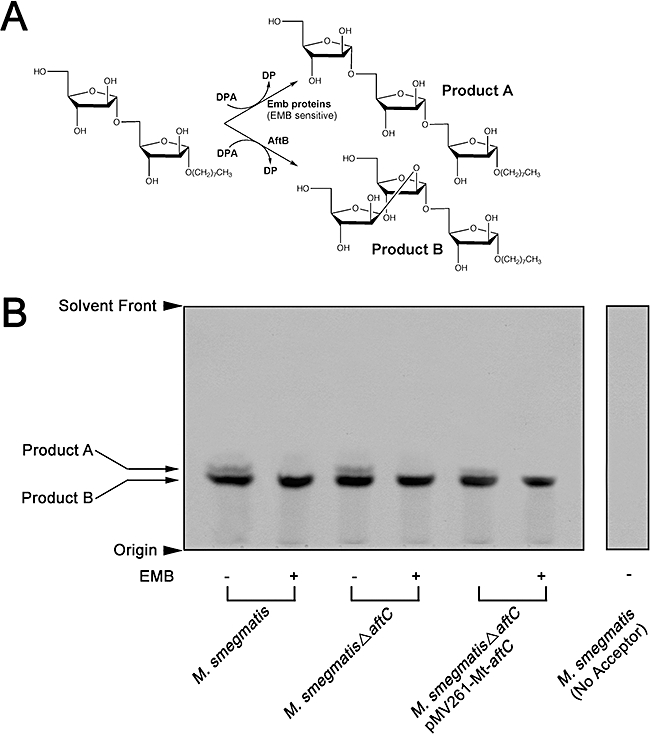
Arabinofuranosyltransferase activity utilizing an Ara_2_ acceptor and membranes prepared from *M. smegmatis*, *M. smegmatis*Δ*aftC* and *M. smegmatis*Δ*aftC* pMV261-Mt-*aftC*. A. Biosynthetic reaction scheme of products A and B formed in arabinofuranosyltransferase assays using the neoglycolipid Ara_2_ acceptor. B. Arabinofuranosyltransferase activity was determined using the synthetic Ara_2_ acceptor in a cell-free assay with and without EMB (1 mg ml^−1^) as previously described (Lee *et al*., 1997). The products of the assay were re-suspended prior to scintillation counting (10%) and the remaining subjected to TLC using silica gel plates (5735 silica gel 60F254, Merck) in CHCl_3_/CH_3_OH/H_2_O/NH_4_OH (65/25/3.6/0.5, v/v/v/v) with the reaction products visualized by autoradiography. The TLC autoradiogram is representative of several independent experiments.

We first assessed whether *M. smegmatis*Δ*aftC* was deficient in α(1→5) and β(1→2) arabinofuranosyltransferase activity using an α-d-Ara*f*-(1→5)-α-d-Ara*f-O*-(CH_2_)_7_CH_3_ (Ara_2_) synthetic acceptor (Lee *et al*., 1997) and DP[^14^C]A as a sugar donor based on an established assay format for determining α(1→5) and β(1→2) arabinofuranosyltransferase activities (Lee *et al*., 1998). TLC/autoradiographic analysis of the products which were only synthesized in the presence of Ara_2_, when assayed with *M. smegmatis* membranes resulted in the formation of two products (A and B) (Fig. 7A and B). The enzymatic synthesis of products A and B are consistent with our previous studies using mycobacterial (Lee *et al*., 1997) and corynebacterial (Seidel *et al*., 2007a) membrane preparations resulting in trisaccharide products as a result of α(1→5) and β(1→2) Ara*f* linkages to the Ara_2_ acceptor (Fig. 7A). Addition of EMB in several experiments, even at high concentrations of up to 1 mg ml^−1^ to the reaction mixture, resulted in a decrease in only the *in vitro* synthesized α-d-[^14^C]Ara*f*-(1→5)-α-d-Ara*f*-(1→5)-α-d-Ara*f-O*-(CH_2_)_7_CH_3_ product A (Fig. 7A and B). Assays performed with membranes from *M. smegmatis*Δ*aftC* and the pMV261-Mt-*aftC* complemented strain using the Ara_2_ synthetic acceptor gave a similar profile to that of wild-type *M. smegmatis* (Fig. 7B). The data clearly show that the *M. smegmatis*Δ*aftC* strain possesses comparable levels of EMB-sensitive α(1→5) and EMB-resistant β(1→2) arabinofuranosyltransferase activity.

**Fig. 8:**
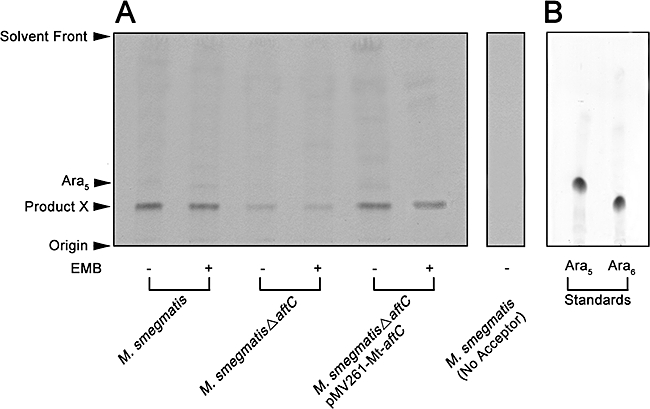
Arabinofuranosyltransferase activity utilizing an Ara_5_ acceptor and membranes prepared from *M. smegmatis*, *M. smegmatis*Δ*aftC* and *M. smegmatis*Δ*aftC* pMV261-Mt-*aftC*. A. Arabinofuranosyltransferase activity was determined using the synthetic Ara_5_ acceptor in a cell-free assay with and without EMB (1 mg ml^−1^). The products reflective of three independent enzyme preparations and assays were re-suspended prior to scintillation counting (10%) and the remaining subjected to TLC using silica gel plates (5735 silica gel 60F254, Merck) in isopropanol/acetic acid/water (8/1/1/, v/v/v) with the reaction product X visualized by autoradiography. The TLC autoradiogram is representative of three independent experiments. B. Ara_5_ and Ara_6_ (Appelmelk *et al*., 2008) acceptor standards were subjected to TLC using silica gel plates (5735 silica gel 60F254, Merck) in isopropanol/acetic acid/water (8/1/1/, v/v/v) with the reaction products visualized by staining with α-naphthol followed by charring.

The lack of α(1→3) arabinofuranosyltransferase activity in the previously reported Ara_2_ and α-d-Ara*f*-(1→5)-α-d-Ara*f*-(1→5)-α-d-Ara*f-O*-(CH_2_)_7_CH_3_ (Ara_3_) acceptor-based assays (Lee *et al*., 1997) required the development of an arabinofuranosyltransferase assay using the Ara-extended synthetic acceptor α-d-Ara*f*-(1→5)-α-d-Ara*f*-(1→5)-α-d-Ara*f*-(1→5)-α-d-Ara*f*-(1→5)-α-d-Ara*f-O*-(CH_2_)_8_NH_2_ (Ara_5_) (*Supplementary experimental* and Fig. S4) and DP[^14^C]A as a sugar donor (Lee *et al*., 1998). TLC/autoradiographic analysis of the products which are only synthesized in the presence of Ara_5_, when assayed with *M. smegmatis* membranes resulted in the formation of a single product X (Fig. 8A) through the transfer of a single [^14^C]Ara*f* residue, with a retardation factor (*R*_*f*_) consistent with a synthetic Ara_6_ acceptor (Appelmelk *et al*., 2008) standard (Fig. 8B). In addition, the synthesis of product X in overexpression studies was enhanced. Consistently from two independent membrane preparations and assays performed in triplicate from *M. smegmatis* pMV261-Mt-*aftC* produced product X (6453 cpm) in comparison to membranes from wild-type *M. smegmatis* (4289 cpm) in the above assays, demonstrating that AftC was functionally involved in the synthesis of product X. The inclusion of EMB in several experiments, even at high concentrations of up to 1 mg ml^−1^ to the reaction mixture did not inhibit the synthesis of this *in vitro* synthesized [^14^C]Ara*f*-Ara_5_ (Fig. 8A, Product X) illustrating that the Ara_5_ acceptor was not extended *via* an EMB-sensitive α(1→5) arabinofuranosyltransferase. Interestingly, membranes prepared from the *M. smegmatis*Δ*aftC* strain were unable to synthesize the *in vitro* product to the same level of activity that was observed with wild-type membranes prepared from *M. smegmatis* (Fig. 8A). This was to be expected, as our earlier *in vivo* and *in vitro* studies would have anticipated residual Ara_6_ product formation, considering that *M. smegmatis*Δ*aftC* possesses β(1→2) arabinofuranosyltransferase activity. Assays performed with membranes from the *M. smegmatis*Δ*aftC* pMV261-Mt-*aftC* complemented strain, gave a similar profile to that of wild-type *M. smegmatis* (Fig. 8A).

**Fig. 9:**
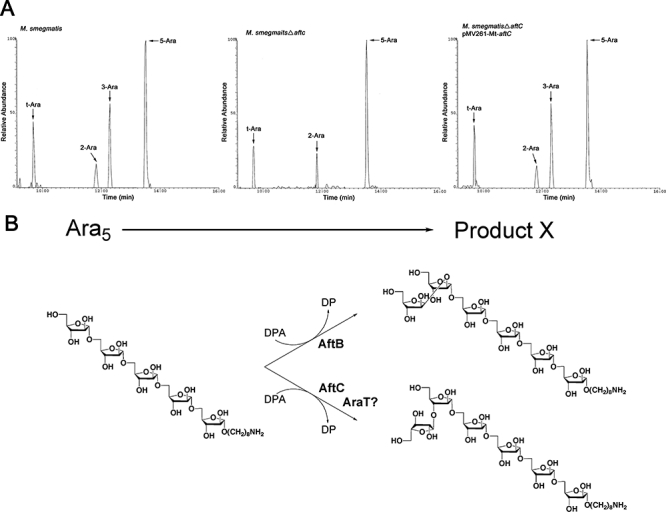
GC characterization of *in vitro* synthesized product X from the arabinofuranosyltransferase assays utilizing the Ara_5_ acceptor. A. GC analysis of the partially per-*O*-methylated, per-*O*-acetylated alditol acetate derivative of product X obtained from assays containing membranes prepared from either *M. smegmatis, M. smegmatis*Δ*aftC* or *M. smegmatis*Δ*aftC* pMV261-Mt-*aftC* (Sassaki *et al*., 2005). B. Panel illustrates the structure(s) of product X.

To establish that the Ara_5_ acceptor is being utilized by two different arabinofuranosyltransferases, presumably establishing β(1→2) and α(1→3) linkages, assays similar to that used before were scaled up (see *Experimental procedures*) and product X extracted and purified through preparative TLC for each membrane preparation. GC (Sassaki *et al*., 2005) and GC/MS (Alderwick *et al*., 2005) analyses of the partially per-*O*-methylated, per-*O*-acetylated alditol acetate derivatives of product X in assays performed with *M. smegmatis* membranes revealed the addition of β(1→2) [R_t_ 11.75 min; *m/z* 129, 130,161,190] and α(1→3) [R_t_ 12.39 min; *m/z* 118, 129, 130, 190, 202, 233] linked Ara*f* residues (Fig. 9A and B). Therefore, the product migrating below Ara_5_ and coincident with the Ara_6_ acceptor standard on TLC (Fig. 8A and B) is in fact a mixture of two products (Fig. 9B). The addition of β(1→2)-linked Ara*f* residues can be attributed to the function of AftB. The presence of α(1→3)-linked Ara*f* residues in this assay using an Ara_5_ acceptor clearly highlights the role of a novel arabinofuranosyltransferase(s) capable of functioning in an α(1→3) capacity. Importantly, the level of α(1→3) activity when the Ara_5_ acceptor is incubated with membranes prepared from *M. smegmatis*Δ*aftC* is completely abolished (Fig. 9A). However, β(1→2) activity is clearly present in *M. smegmatis*Δ*aftC* (Fig. 9A). In addition, *M. smegmatis*Δ*aftC* complemented with pMV261-Mt-*aftC* restores α(1→3) arabinofuranosyltransferase activity to wild-type *M. smegmatis* (Fig. 9A). The results clearly establish both from *in vivo* and *in vitro* experiments that AftC catalyses the addition of an α(1→3)-Ara*f* unit *via* an α(1→3) arabinofuranosyltransferase and that this enzyme is also resistant to EMB (Fig. 8A).

## Discussion

The mAGP complex represents one of the most important cell wall components of the *Corynebacterianeae* and is essential for the viability of *M. tuberculosis* (Vilcheze *et al*., 2000; Pan *et al*., 2001; Gande *et al*., 2004; Mills *et al*., 2004). It is therefore not surprising that one of the most effective antimycobacterial drugs, EMB, targets its synthesis through inhibition of AG biosynthesis. However, the emergence of MDR-TB and XDR-TB has accelerated the need to discover new drug targets (Brennan and Nikaido, 1995). One of the strategies is to identify genes involved in AG biosynthesis. Based on this strategy we previously identified the presence of a new ‘priming’ enzyme, now termed AftA, which would link the initial Ara*f* unit with the C-5 OH of a β(1→6) linked Gal*f* of a pre-synthesized galactan core (Alderwick *et al*., 2005), and more recently identified the AftB enzyme responsible for β(1→2) Ara*f* residues.

The previously described Emb (Alderwick *et al*., 2005), AftA (Alderwick *et al*., 2006a) and AftB proteins (Seidel *et al*., 2007a) are distinct arabinofuranosyltransferases. Thus, despite some functional relationship, these glycosyltransferases have inherent specific features as evident from the insensitivity of AftA and AftB towards EMB, whereas the single Cg-Emb (Alderwick *et al*., 2005; Radmacher *et al*., 2005) and Mt-Emb proteins are sensitive towards EMB (Telenti *et al*., 1997; Belanger *et al*., 1996). The number of arabinofuranosyltransferases that are required for mycobacterial arabinan biosynthesis has been a matter of speculation to date depending on how the arabinan chains are assembled. The primary structure of AG (Besra *et al*., 1995; Daffé *et al*., 1990) would suggest at least five distinct arabinofuranosyltransferases are required for the complete formation of AG. Interestingly, *M. smegmatis embA* and *embB* mutants were found to possess reduced amounts of the non-reducing terminal disaccharide β-d-Ara*f*-(1→2)-α-d-Ara*f* and result in the removal of the dominant terminal non-reducing Ara_6_ branched motif in the mutant being replaced by a linear Ara_4_ motif (Escuyer *et al*., 2001). The authors of this study concluded that the *M. smegmatis embA* and *embB* mutants result in a lack of 3-arm branching off the main α(1→5)-arabinan chain proximal to the non-reducing and attachment site of mycolic acids in AG (Escuyer *et al*., 2001). Initially, it was proposed that the β-d-Ara*f*-(1→2)-α-d-Ara*f* disaccharide was assembled using EmbA and EmbB. However, the recent identification of AftB, the development of specific *in vitro* assays in combination with mutant strains, and recent structural studies have fuelled speculation that EmbA/B act as α(1→5) arabinofuranosyltransferases (Alderwick *et al*., 2005; Seidel *et al*., 2007a; Bhamidi *et al*., 2008).

**Fig. 10:**
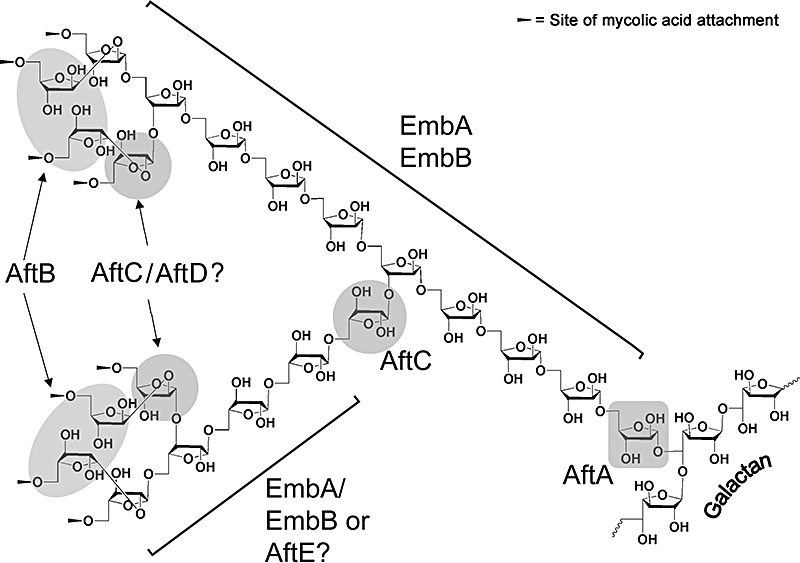
Mycobacterial arabinan biosynthesis and the role of AftC.

In this study, we have identified MSMEG2785 (also Rv2673 and NCgl1822, which we have termed AftC, as a novel branching arabinofuranosyltransferase. More precisely, AftC catalyses the addition of α(1→3) Ara*f* residues as shown through both *in vivo* and *in vitro* experiments, ultimately resulting in 3,5-Ara*f* residues after further α(1→5) extension, characteristic of AG. For instance, incubation of membranes prepared from *M. smegmatis* with DP[^14^C]A and a linear α(1→5)-Ara_5_ neoglycolipid acceptor resulted in the synthesis of an Ara_6_ product. Further chemical characterization of the product by glycosyl linkage analysis established that the α(1→5)-Ara_5_ acceptor was extended *via* an EMB-resistant α(1→3) arabinofuranosyltransferase giving rise to 3-linked Ara*f* residues and corroborated our earlier cell wall analysis of the *M. smegmatis*Δ*aftC* mutant. As it is now established that only α(1→5) arabinofuranosyltransferase(s) is EMB-sensitive it can be further speculated that EmbA and EmbB function in the assembly of the linear α(1→5) arabinan segments as presented in Fig. 10, which is in accordance with previous data and the phenotype of a Cg-Emb mutant (Alderwick *et al*., 2005). It is clear that further studies are required to establish the precise role of EmbA and EmbB in mycobacteria.

The analysis of the *M. smegmatis*Δ*aftC* mutant to date and based on the Ara : Gal ratio would suggest that the residual arabinan segment in the mutant consists of approximately five Ara*f* residues: β-d-Ara*f*-(1→2)-α-d-Ara*f*-(1→5)-α-d-Ara*f*-(1→5)-α-d-Ara*f*-(1→5)-α-d-Ara*f* located at three branches on the galactan chain (Besra *et al*., 1995; Alderwick *et al*., 2005). This is consistent with the recent primary structure of AG (Bhamidi *et al*., 2008), with a ‘non-variable’ terminal non-reducing Ara_17_ motif, introduction of a 3,5-Ara*f* residue distal to this non-reducing end by AftC and further extension by a linear α(1→5)Ara*f* domain (Fig. 10). The latter appears to be variable (up to 12/13 residues). However, based on *M. smegmatis*Δ*aftC* and the subsequent Ara : Gal compositional analysis a dominant Ara_22_/Ara_23_ motif would be consistent with recent (Bhamidi *et al*., 2008) and previous (Besra *et al*., 1995) structural data on AG and this is represented in terms of biosynthetic considerations in Fig. 10. It is also possible that AftC or a second distinct α(1→3) arabinosfuranosyltransferase (shown as AftD in Fig. 10) may be involved in late stages of AG synthesis, i.e. the non-reducing Ara_6_ motif and is consistent with our data and the model presented in Fig. 10.

The discovery of AftC has now shed new light on the key arabinofuranosyltransferases to build an arabinan domain typical for *Corynebacterianae*. In this context, the genomic organization in the genomes of the *Corynebacterianae* sequenced is intriguing, revealing high synteny of the *M. tuberculosis aftC* locus to the maps of all other *Mycobacterium* and *Corynebacterium* species. The identification of new cell wall biosynthetic drug targets is of great importance, especially with the emergence of MDR-TB. This newly discovered DPA-dependent arabinofuranosyl transferase represents, along with a straightforward *in vitro* enzyme assay, a promising candidate for further exploitation as a potential drug target.

## Experimental procedures

### Bacterial strains and growth conditions

*Corynebacterium glutamicum* ATCC 13 032 (referred to the remainder of the text as *C. glutamicum*) and *Escherichia coli* DH5αmcr were grown in Luria–Bertani broth (LB, Difco) at 30°C and 37°C respectively. The recombinant strains generated in this study were grown on complex brain–heart infusion medium (BHI, Difco), and the salt medium CGXII used for *C. glutamicum* as described (Eggeling and Reyes, 2005). Kanamycin and ampicillin were used at a concentration of 50 μg ml^−1^. *M. smegmatis* strains were grown in tryptic soy broth (TSB; Difco), containing 0.005% Tween80 (TSBT). Solid media were made by adding 1.5% agar to the above-mentioned broths. The concentrations of antibiotics used for *M. smegmatis* were 100 μg ml^−1^ for hygromycin and 20 μg ml^−1^ for kanamycin. Minimal inhibitory concentrations were determined by plating cells on solid media supplemented with various concentrations of EMB, rifampicin and chloramphenicol. The minimal inhibitory concentration was defined as the first concentration of drug that would inhibit 100% of growth after 5 days of incubation (Belanger *et al*., 1996). *M. tuberculosis* H37Rv DNA was obtained from the NIH Tuberculosis Research Materials and Vaccine Testing Contract at Colorado State University. All other chemicals were of reagent grade and obtained from Sigma-Aldrich.

### Construction of plasmids and strains

Approximately 1 kb of upstream and downstream flanking sequences of *MSMEG2785* were PCR amplified from *M. smegmatis* mc^2^155 genomic DNA using the primer pairs MS2785LL (TTTTTTTTCCATAAATTGGATCCGCTGACCGACCTCATC) and MS2785LR (TTTTTTTTCCATTTCTTGGCGAGCCCGAGCTTGAAGTTG), and MS2785RL (TTTTTTTTCCATAGATTGGTTCCTGCTGCTGTCCCTTGG) and MS2785RR (TTTTTTTTCCATCTTTTGGCGAACTCAGCGGCGATTCAC) respectively (all primers are given in 5′ to 3′ direction). Following restriction digestion of the primer incorporated *Van*91I sites, the PCR fragments were cloned into *Van*91I-digested p0004S to yield the knockout plasmid pΔ*MSMEG2785* which was then packaged into the temperature-sensitive mycobacteriophage phAE159 as described previously (Bardarov *et al*., 2002) to yield phasmid DNA of the knockout phage phΔ*MSMEG2785*. Generation of high titre phage particles and specialized transduction were performed as described earlier (Stover *et al*., 1991; Bardarov *et al*., 2002). Deletion of *MSMEG2785* in one hygromycin-resistant transductant was confirmed by Southern blot. To enable expression of *MSMEG2785* and *Rv2673*, in the deletion mutant, these were amplified using primer pairs designed for subsequent cloning into the mycobacterial-shuttle vector pMV261 (Stover *et al*., 1991). All cloned fragments were verified by sequencing.

To construct the deletion vector pK19mobsacBΔ*aftC* (NCgl 1822), cross-over PCR was applied with primer pairs AB (A, CGTTAAGCTTCGATCTTGTTGATGTGTGGCATCACACG; B, CCCATCCACTAAACTTAAACAGCGCCATCAACAACATGG) and CD (C, TGTTTAAGTTTAGTGGATGGGTGATCCAACGCACGACCATC; D, GCATGGATCCACGCATACCGAGGGAAAGATCTTC) and *C. glutamicum* genomic DNA as template. Both amplified products were used in a second PCR with primer pairs AD to generate a 656 bp fragment consisting of sequences adjacent to Cg-*aftC*, which was ligated with BamHI–HindIII-cleaved pK19mobsacB. All plasmids were confirmed by sequencing. The chromosomal deletion of Cg-*aftC* was performed as described previously using two rounds of positive selection (Schafer *et al*., 1994), and its successful deletion was verified by use of two different primer pairs.

### Isolation of the mAGP complex, glycosyl composition and linkage analysis of alditol acetates by GC and GC/MS

The thawed cells were re-suspended in phosphate-buffered saline containing 2% Triton X-100 (pH 7.2), disrupted by sonicaton and centrifuged at 27 000 *g* (Besra *et al*., 1995; Alderwick *et al*., 2005). The pelleted material was extracted three times with 2% SDS in phosphate-buffered saline at 95°C for 1 h, washed with water, 80% (v/v) acetone in water and acetone, and finally lyophilized to yield a highly purified cell wall preparation (Besra *et al*., 1995; Alderwick *et al*., 2005). Cell wall or per-*O*-methylated cell wall preparations (Alderwick *et al*., 2005) were hydrolysed in 2 M TFA, reduced with NaB^2^H_4_ and the resultant alditols per-*O*-acetylated and examined by GC and GC/MS as described previously (Besra *et al*., 1995; Alderwick *et al*., 2005).

### Extraction and analysis of cell wall-bound mycolic acids

In terms of *M. smegmatis* strains equivalent amounts of freeze-dried bacilli (100 mg) were processed as described previously (Seidel *et al*., 2007a), following two consecutive CHCl_3_/CH_3_OH/H_2_O (10:10:3, v/v/v) extractions for 4 h at 50°C in the analysis of cell wall-associated lipids, and cell wall-bound MAMEs. Alternatively, *M. smegmatis* and *C. glutamicum* cultures (5 ml) were grown and metabolically labelled at mid-logarithmic phase of growth using 1 μCi ml^−1^ [1,2-^14^C]acetate (50–62 mCi mmol^−1^, GE Healthcare, Amersham Bioscience) for 4 h at either 30°C or 37°C with gentle shaking, harvested, washed and freeze-dried. Cells were then extracted by two consecutive extractions with 2 ml of CHCl_3_/CH_3_OH/H_2_O (10:10:3, v/v/v) for 4 h at 50°C to provide cell wall-associated lipids and analysed as described previously (Seidel *et al*., 2007a). The crude lipid extracts were re-suspended in CHCl_3_/CH_3_OH (2:1) and equivalent aliquots (50 000 cpm) analysed by TLC using silica gel plates (5735 silica gel 60F254, Merck) developed in CHCl_3_/CH_3_OH/NH_4_OH (80:20:2, v/v/v) to separate [^14^C]-labelled TDM and phospholipids (Mikusova *et al*., 1995). Lipids were visualized by autoradiography by overnight exposure of Kodak X-Omat AR film to the TLC plates to reveal labelled lipids, quantified by phosphorimaging and compared with know standards (Mikusova *et al*., 1995). The bound MAMEs/CMAMEs from the above de-lipidated extracts were released by the addition of 2 ml of 5% aqueous solution of tetra-butyl ammonium hydroxide followed by overnight incubation at 100°C. After cooling, water (2 ml), CH_2_Cl_2_ (4 ml) and CH_3_I (500 μl) were added and mixed thoroughly for 30 min. The lower organic phase was recovered following centrifugation and washed three times with water (4 ml), dried and re-suspended in diethyl ether (4 ml). After centrifugation the clear supernatant was again dried and re-suspended in CH_2_Cl_2_ (100 μl). An aliquot (5 μl) from each strain was subjected to scintillation counting and an equivalent (5 μl) aliquot analysed by TLC using silica gel plates (5735 silica gel 60F254, Merck), developed in petroleum ether/acetone (95:5, v/v) and either visualized by autoradiography by exposure of Kodak X-Omat AR film to the TLC plates to reveal [14C]-labelled MAMEs/CMAMEs, or charred following spraying with 5% molybdophosphoric acid in ethanol at 100°C and compared with know standards.

### Arabinofuranosyltransferase activity with membrane preparations of *M. smegmatis*, *M. smegmatis* pMV261-Mt-*aftC*, *M. smegmatisΔaftC* and *M. smegmatisΔaftC* pMV261-Mt-*aftC*

Membranes were prepared as described previously (Lee *et al.,* 1997; Alderwick *et al*., 2006a) and re-suspended in 50 mM MOPS (pH 7.9), containing 5 mM β-mercaptoethanol and 10 mM MgCl_2_ (buffer A) to a final concentration of 15–10 mg ml^−1^. The neoglycolipid acceptors used in this study were α-d-Ara*f*-(1→5)-α-d-Ara*f*-(1→5)-α-d-Ara*f*-(1→5)-α-d-Ara*f*-(1→5)-α-d-Ara*f-O*-(CH_2_)_8_NH_2_ (Ara_5_, see Supplementary material) and α-d-Ara*f*-(1→5)-α-d-Ara*f-O*-(CH_2_)_7_CH_3_ (Ara_2_) (Lee *et al*., 1995; 1998). The acceptors (either Ara_2_ or Ara_5_) and DP[^14^C]A (Lee *et al*., 1995; 1998) (stored in CHCl_3_/CH_3_OH, 2:1, v/v) were aliquoted into 1.5 ml eppendorf tubes to a final concentration of 2 mM and 200 000 cpm (90 μM), respectively, and dried under nitrogen. The arabinofuranosyltransferase assay was carried out as described previously (Lee *et al*., 1997) with modifications. IgePal™ (Sigma-Aldrich) was added (0.1%, v/v) with the appropriate amount of buffer A (final volume 80 μl). Tubes were sonicated for 15 min to re-suspend lipid linked substrates and then mixed with the remaining assay components, which included membrane protein from either *M. smegmatis*, *M. smegmatis* pMV261-Mt*-aftC M. smegmatis*Δ*aftC* or *M. smegmatis*Δ*aftC* pMV261-Mt-*aftC* (1 mg), 1 mM ATP, 1 mM NADP and in some cases EMB (0–1 mg ml^−1^). Assays were incubated for 1 h at 37°C and quenched by the addition of 533 μl CHCl_3_/CH_3_OH (1:1, v/v). After mixing and centrifugation at 27 000 *g* for 15 min at 4°C, the supernatant was removed and dried under nitrogen. The residue was then re-suspended in 700 μl of CH_3_CH_2_OH/H_2_O (1:1, v/v) and loaded onto a 1 ml SepPak strong anion exchange cartridge (Supelco), pre-equilibrated with CH_3_CH_2_OH/H_2_O (1:1, v/v). The column was washed with 2 ml CH_3_CH_2_OH and the eluate collected, dried and partitioned between the two phases arising from a mixture of *n*-butanol (3 ml) and water (3 ml). The resulting organic phase was recovered following centrifugation at 3500 *g* and the aqueous phase again extracted twice with 3 ml of water-saturated *n*-butanol. The pooled extracts were back-washed twice with *n*-butanol-saturated water (3 ml). The *n*-butanol fraction was dried and re-suspended in 200 μl butanol. The extracted radiolabelled material was quantified by liquid scintillation counting using 10% of the labelled material and 5 ml of EcoScintA (National Diagnostics, Atlanta). The incorporation of [^14^C]Ara*f* was determined by subtracting counts present in control assays (incubations in the absence of acceptor). The remaining labelled material was subjected to thin-layer chromatography (TLC) using either isopropanol/acetic acid/water (8:1:1, v/v/v) for the assays utilizing the Ara_5_ acceptor or CHCl_3_/CH_2_OH/H_2_O/NH_4_OH (65:25:3.6:0.5, v/v/v/v) in the case of the Ara_2_ acceptor on aluminum-backed Silica Gel 60 F254 plates (Merck, Darmstadt, Germany). Autoradiograms were obtained by exposing TLCs to X-ray film (Kodak X-Omat) for 3 days.

### Characterization of α(1→3)-arabinofuranosyltransferase activity with membranes prepared from *M. smegmatis, M. smegmatisΔaftC* and *M. smegmatisΔaftC* pMV261-Mt-*aftC*

Large-scale reaction mixtures containing cold DPA (200 μg, 0.75 mM) (Lee *et al*., 1997) and 50 mM of the acceptor Ara_5_ were mixed and given an initial incubation at 37°C with membranes prepared from either *M. smegmatis*, *M. smegmatis*Δ*aftC* or *M. smegmatisΔaftC* pMV261-Mt-*aftC* for 1 h. The assays were replenished with fresh membranes (1 mg) and re-incubated for 1 h at 37°C with the entire process repeated thrice. Products were extracted from reaction mixtures by *n*-butanol/water phase separation as described earlier to extract products. Products were applied to preparative TLC plates, developed in isopropanol/acetic acid/water (8:1:1, v/v/v) and sprayed with 0.01% 1,6-diphenylhexatriene in petroleum-ether/acetone (9:1, v/v), and the products localized under long-wave (366 nm) UV light (Lee *et al*., 1997). The plate was then re-developed in toluene to remove the reagent and the bands recovered from the plates by extraction with *n*-butanol. The butanol phases were washed with water saturated with *n*-butanol and the dried products subjected to GC (Sassaki *et al*., 2005) and GC/MS as described (Lee *et al*., 1997; Alderwick *et al*., 2006a).

## Supplementary material

This material is available as part of the online article from: http://www.blackwell-synergy.com/doi/abs/10.1111/

j.1365-2958.2008.06354.x

(This link will take you to the article abstract).
