# Supplementary material for: Biosynthesis of mycobacterial arabinogalactan: identification of a novel α(1→3) arabinofuranosyltransferase
Source: Mol Microbiol. 2008 Jul 15;69(5):1191–206. doi: 10.1111/j.1365-2958.2008.06354.x (PMC2610374; doi:10.1111/j.1365-2958.2008.06354.x)
Supplement: Supplementary file 1 [file mmi_6354_SD1.pdf]

Supplementary Material

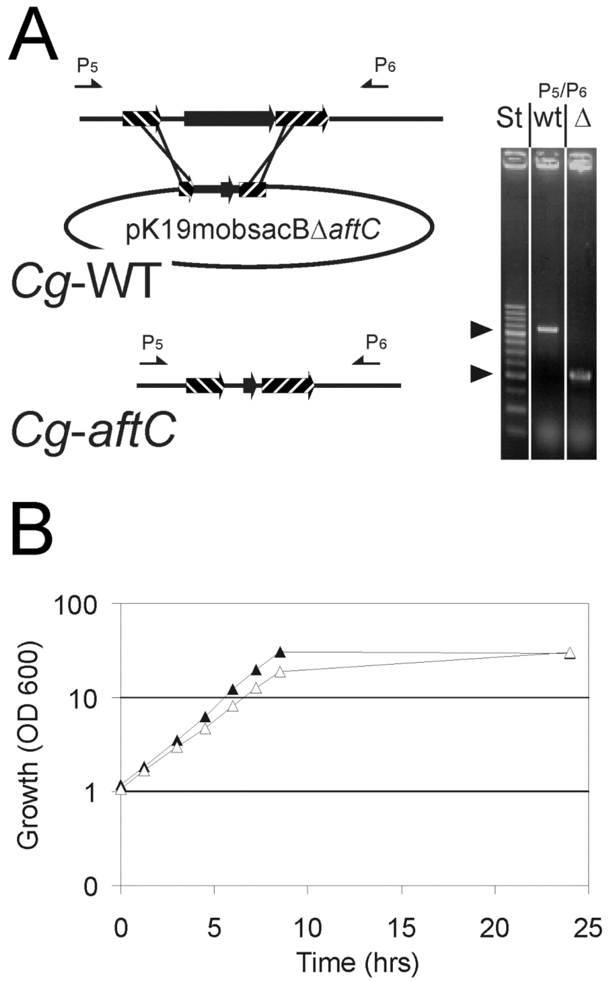

Fig. S1. Strategy to delete Cg-*aftC* using the deletion vector pK19mobsacBΔ*aftC*. (A) This vector carries 18 nucleotides of the 5' end of Cg-*aftC* and 36 nucleotides of its 3' end thereby enabling the in-frame deletion of almost the entire Cg-*aftC* gene. The arrows marked P5 and P6 locate the primers used for the PCR analysis to confirm the absence of Cg-*aftC*. Distances are not drawn to scale. The results of the PCR analysis with the primer pair P5/P6 are shown on the right. Amplification products obtained from the wild type (wt) were applied in the middle lane and that of the deletion mutant in the right lane. 'St' marks the standard, where the arrowheads are located at 1 and 0.5 kb. **(B)** Growth of the wild type of *C. glutamicum* (▲) and *C. glutamicum*Δ*aftC* (△) on salt medium CGXII with glucose as carbon source.

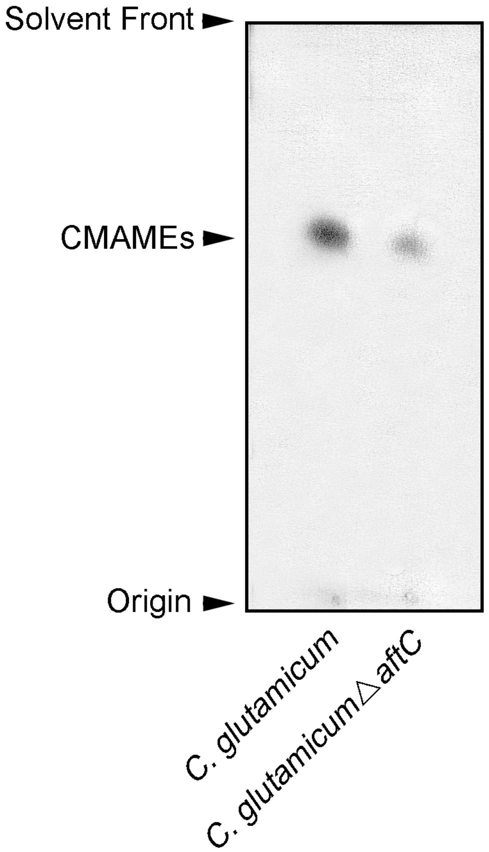

Fig. S2. Analysis of cell wall bound CMAMEs from *C. glutamicum* and *C. glutamicun∆aftC*. The bound [^{14}C]-labeled corynemycolic acids from de-lipidated [^{14}C]-labeled cell of pulse-labeled 5 ml cultures were released by the addition of tetra-butyl ammonium hydroxide at 100°C overnight and methylated as described in the "Experimental Procedures". An equivalent aliquot from each strain was subjected to TLC/autoradiography using silica gel plates (5735 silica gel 60F_{254}, Merck), and developed in petroleum ether/acetone (95:5, v/v) reveal CMAMEs and compared to known standards.

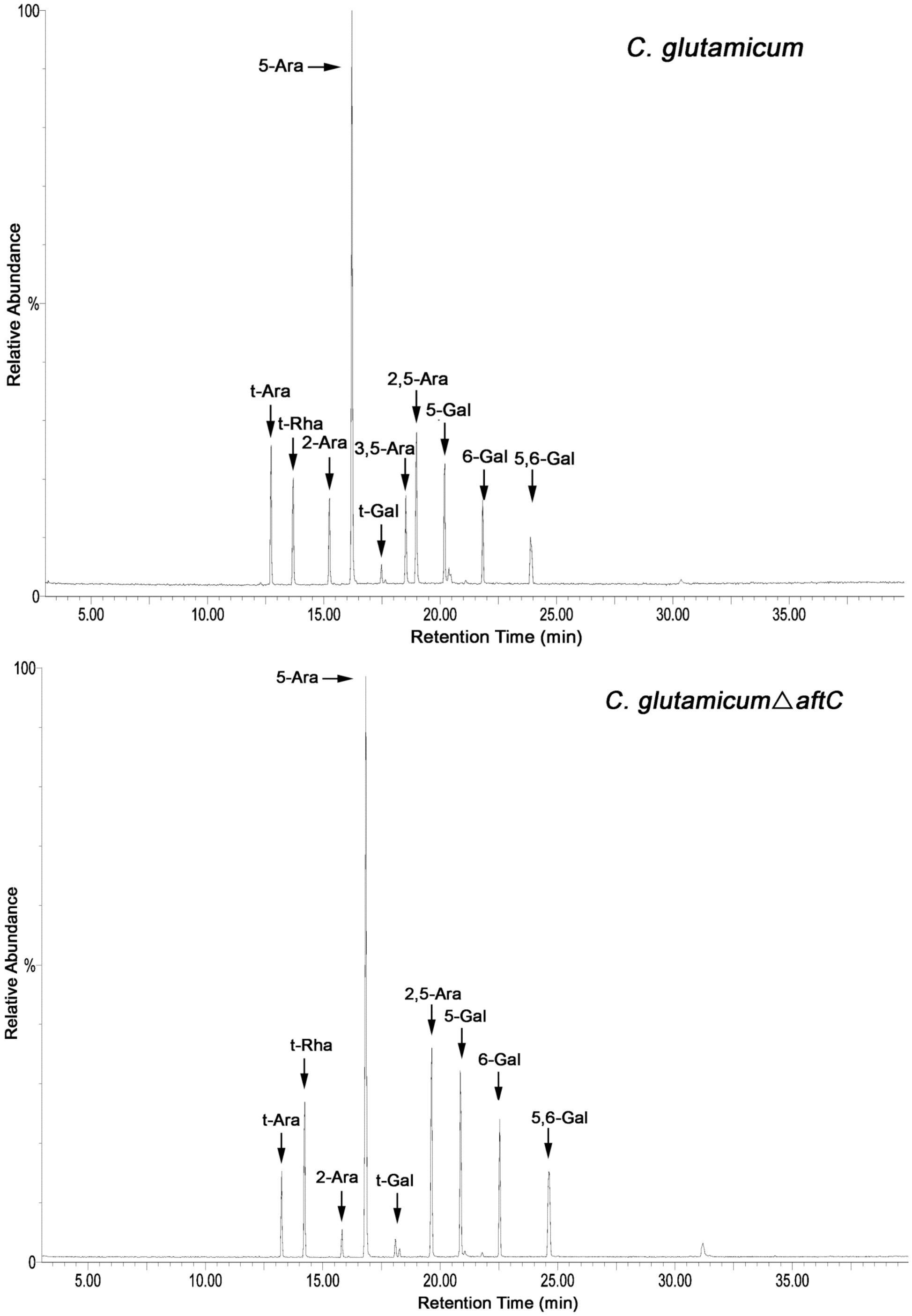

Fig. S3. GC/MS analysis of cell walls of *C. glutamicum* and *C. glutamicum*Δ*aftC*. Samples of per-*O*-methylated cell walls were, hydrolyzed using 2M TFA, reduced, per-*O*-acetylated and analyzed as described under “Experimental Procedures” .

Experimental

General Methods. Reactions were carried out in oven-dried glassware. All reagents used were purchased from commercial sources and were used without further purification unless noted. Reaction solvents were purified by successive passage through columns of alumina and copper under nitrogen. Unless stated otherwise, all reactions were carried out at room temperature under a positive pressure of argon and were monitored by TLC on Silica Gel 60 F_{254} (0.25 mm, E. Merck). Spots were detected under UV light or by charring with acidified *p*-anisaldehyde solution in EtOH. Unless otherwise indicated, all column chromatography was performed on Silica Gel (40–60 μM). The ratio between silica gel and crude product ranged from 100 to 50:1 (w/w). Optical rotations were measured at 22 ± 2 °C. ^{1}H NMR spectra were recorded at 600 MHz, 500 MHz or 400 MHz, and chemical shifts were referenced to either TMS (0.0, $CDCl_3$) or external acetone (2.22, D_2O). ^{1}H data were reported as though they were first order. ^{13}C NMR (APT) spectra were recorded at 125 MHz or 100 MHz, and ^{13}C chemical shifts were referenced to internal $CDCl_3$ (77.23, $CDCl_3$) or external acetone (31.07, D_2O). Assignments of resonances in NMR spectra were done using 1H-1H COSY and HMQC experiments. Organic solutions were concentrated under vacuum at < 40 °C. Electrospray mass spectra were recorded on samples suspended in mixtures of THF with CH_3OH and added NaCl.

8-Azidooctyl 2,3-Di-*O*-benzoyl-5-*O*-*tert*-butyldiphenylsilyl-α-D-arabinofuranosyl-(1→5)-2,3-di-*O*-benzoyl-α-D-arabinofuranoside (3)

Alcohol **1**[1] (0.681 g, 1.33 mmol) and thioglycoside **2**[2] (1.029 g, 1.46 mmol) were dried over P_2O_5 under vacuum for 6 h and then dissolved in CH_2Cl_2 (25 ml) and the resulting solution was cooled to 0 °C. Powdered 4 Å molecular sieves (0.5 g) were added and the suspension was stirred for 30 min at 0 °C before *N*-iodosuccinimide (0.381 g, 1.61 mmol) and silver triflate (0.041 g, 0.16

mmol) were added. The reaction mixture was stirred for 15 min, neutralized with Et_3N, diluted with CH_2Cl_2 (25 ml) and filtered through Celite. The filtrate was washed successively with a saturated aqueous $Na_2S_2O_3$ solution (30 ml × 2) and water (30 ml) before being dried (Na_2SO_4) and concentrated. The crude residue was purified by column chromatography (6:1, hexanes/EtOAc) to afford **3** (1.266 g, 87%) as a white foam. R_f 0.37 (4:1, hexanes/EtOAc); $[\alpha]_D$ +4.4 (*c* 0.7, CH_2Cl_2); ^{1}H NMR (400 MHz, $CDCl_3$, δ_H) 8.13–7.97 (m, 8 H, Ar), 7.79–7.73 (m, 4 H, Ar), 7.63–7.32 (m, 18 H, Ar), 5.72–5.67 (m, 2 H, H-3, H-3'), 5.65 (s, 1 H, H-2), 5.57 (s, 1 H, H-2'), 5.44 (s, 1 H, H-1'), 5.28 (s, 1 H, H-1), 4.60 (ddd, 1 H, *J* = 4.6, 4.6, 4.5 Hz, H-4'), 4.55–4.51 (m, 1 H, H-4), 4.26 (dd, 1 H, *J* = 11.1, 4.6 Hz, H-5), 4.08–4.02 (m, 2 H, H-5' × 2), 4.00 (dd, 1 H, *J* = 11.1, 2.8 Hz, H-5), 3.82 (ddd, 1 H, *J* = 9.5, 6.6, 6.6 Hz, octyl OCH_2), 3.56 (ddd, 1 H, *J* = 9.5, 6.4, 6.4 Hz, octyl OCH_2), 3.24 (dd, 2 H, *J* = 6.9, 6.9 Hz, octyl CH_2N_3), 1.74–1.54 (m, 4 H, octyl CH_2), 1.50–1.25 (m, 8 H, octyl CH_2), 1.18 (s, 9 H, *tert*-butyl $C(CH_3)_3$); ^{13}C NMR (100 MHz, $CDCl_3$, δ_C) 165.6 (C=O), 165.4 (C=O), 165.3 (C=O), 165.1 (C=O), 135.6 (Ar), 135.5(8) (Ar), 133.3 (Ar), 133.2 (Ar), 133.1 (Ar), 133.0 (Ar), 129.9 (Ar), 129.8 (Ar), 129.7(7) (Ar), 129.7 (Ar), 129.6 (Ar), 129.4 (Ar), 129.3 (Ar), 129.2 (Ar), 129.0 (Ar), 128.4 (Ar), 128.3(5) (Ar), 128.3 (Ar), 128.2 (Ar), 127.6 (Ar), 105.9 (C-1'), 105.6 (C-1), 83.2 (C-4'), 82.1 (C-2), 81.8 (C-2'), 81.7 (C-4), 77.4 (C-3'), 77.3 (C-3), 67.3 (octyl OCH_2), 66.1 (C-5), 63.4 (C-5'), 51.3 (octyl CH_2N_3), 29.4 (octyl CH_2), 29.2 (octyl CH_2), 29.0 (octyl CH_2), 28.7 (octyl CH_2), 26.7 (*tert*-butyl $C(\mathit{C}H_3)_3$), 26.6 (octyl CH_2), 26.0 (octyl CH_2), 19.2 (*tert*-butyl $\mathit{C}(CH_3)_3$); HRMS (ESI) calcd for (M+Na) $C_{62}H_{67}N_3O_{13}SiNa$: 1112.4335, found 1112.4332.

8-Azidooctyl 2,3-Di-*O*-benzoyl-α-D-arabinofuranosyl-(1→5)-2,3-di-*O*-benzoyl-α-D-arabinofuranoside (4)

To a solution of compound **3** (1.11 g, 1.02 mmol) in pyridine-THF (1:3 v:v, 12 ml) at 0 °C was added HF-pyridine (0.5 ml) dropwise. The reaction mixture was warmed to room temperature and stirred for 16 h. The reaction mixture was diluted with EtOAc (20 ml), poured into a saturated aq. $NaHCO_3$ soln (60 ml), and extracted with EtOAc (30 ml × 2). The combined organic layer was washed with water (60 ml), dried (Na_2SO_4) and concentrated to give a crude syrup that was purified by column chromatography (3:1, hexanes/EtOAc) to afford **4** (0.787 g, 91%) as a white foam. R_f 0.23 (3:1, hexanes/EtOAc); $[\alpha]_D$ -1.7 (*c* 0.4, CH_2Cl_2); ^{1}H NMR (500 MHz, $CDCl_3$, δ_H) 8.08–8.02 (m, 6 H, Ar), 7.97–7.93 (m, 2 H, Ar), 7.62–7.38 (m, 10 H, Ar), 7.32–7.28 (m, 2 H, Ar), 5.66 (d, 1 H, *J* = 1.3 Hz, H-2'), 5.61 (dd, 1 H, *J* = 4.8, 1.2 Hz, H-3), 5.51 (d, 1 H, *J* = 1.2 Hz, H-2), 5.44 (dd, 1 H, *J* = 4.4, 1.3 Hz, H-3'), 5.42 (s, 1 H, H-1'), 5.23 (s, 1 H, H-1), 4.50 (ddd, 1 H, *J* = 4.4, 4.4, 4.0 Hz, H-4'), 4.44 (ddd, 1 H, *J* = 4.8, 4.8, 3.0 Hz, H-4), 4.20 (dd, 1 H, *J* = 11.2, 4.8 Hz, H-5), 4.03 (dd, 1 H, *J* = 12.1, 4.4 Hz, H-5'), 3.99–3.94 (m, 2 H, H-5, H-5'), 3.75 (ddd, 1 H, *J* = 9.5, 6.7, 6.7 Hz, octyl OCH_2), 3.51 (ddd, 1 H, *J* = 9.5, 6.3, 6.3 Hz, octyl OCH_2), 3.22 (dd, 2 H, *J* = 7.0, 7.0 Hz, octyl CH_2N_3), 2.24 (br s, 1 H, OH), 1.68–1.53 (m, 4 H, octyl CH_2), 1.44–1.24 (m, 8 H, octyl CH_2); ^{13}C NMR (125 MHz, $CDCl_3$, δ_C) 166.1 (C=O), 165.7 (C=O), 165.4 (C=O), 165.1 (C=O), 133.5 (Ar), 133.4(4) (Ar), 133.4 (Ar), 133.3 (Ar), 129.9 (Ar), 129.8(6) (Ar), 129.8(2) (Ar), 129.8 (Ar), 129.3 (Ar), 129.2 (Ar), 129.1 (Ar), 129.0 (Ar), 128.5 (Ar), 128.4 (Ar), 128.3 (Ar), 105.8 (C-1'), 105.6 (C-1), 83.7 (C-4'), 81.8(5) (C-4), 81.8 (C-2), 81.7 (C-2'), 77.8 (C-3'), 77.4 (C-3), 67.4 (octyl OCH_2), 66.2 (C-5), 62.3 (C-5'), 51.4 (octyl CH_2N_3), 29.5 (octyl CH_2), 29.2 (octyl CH_2), 29.1 (octyl CH_2), 28.8 (octyl CH_2), 26.6 (octyl CH_2), 26.1 (octyl CH_2); HRMS (ESI) calcd for (M+Na) $C_{46}H_{49}N_3O_{13}Na$: 874.3157, found 874.3154.

8-Azidooctyl 2,3-Di-*O*-benzoyl-5-*O*-*tert*-butyldiphenylsilyl-α-D-arabinofuranosyl-(1→5)-2,3-di-*O*-benzoyl-α-D-arabinofuranosyl-(1→5)-2,3-di-*O*-benzoyl-α-D-arabinofuranosyl-(1→5)-2,3-di-*O*-benzoyl-α-D-arabinofuranoside (6)

Prepared from thioglycoside **5**[1] (0.880 g, 0.844 mmol), alcohol **4** (0.599 g, 0.703 mmol), 4 Å molecular sieves (0.5 g), *N*-iodosuccinimide (0.228 g, 0.963 mmol) and silver triflate (0.030 g, 0.012 mmol) in CH_2Cl_2 (20 ml) as described for **3**, to afford **6** (1.16 g, 93%) as a syrup. R_f 0.38 (3:1, hexanes/EtOAc); $[\alpha]_D$ +14.7 (*c* 0.5, CH_2Cl_2); ^{1}H NMR (600 MHz, $CDCl_3$, δ_H) 8.08–7.91 (m, 16 H, Ar), 7.73–7.70 (m, 4 H, Ar), 7.60–7.24 (m, 30 H, Ar), 5.69–5.64 (m, 6 H), 5.59 (s, 1 H), 5.54 (s, 1 H), 5.43 (s, 1 H, H-1), 5.42 (s, 1 H, H-1), 5.40 (s, 1 H, H-1), 5.24 (s, 1 H, H-1), 4.66–4.62 (m, 2 H), 4.52 (ddd, 1 H, *J* = 4.7, 4.7, 4.5 Hz), 4.48–4.44 (m, 1 H), 4.24–4.18 (m, 3 H), 4.02-3.93 (m, 5 H), 3.77 (ddd, 1 H, *J* = 9.5, 6.8, 6.8 Hz, octyl OCH_2), 3.52 (ddd, 1 H, *J* = 9.5, 6.3, 6.3 Hz, octyl OCH_2), 3.23 (dd, 2 H, *J* = 6.9, 6.9 Hz, octyl CH_2N_3), 1.70–1.54 (m, 4 H, octyl CH_2), 1.44–1.26 (m, 8 H, octyl CH_2), 1.04 (s, 9 H, *tert*-butyl $C(CH_3)_3$); ^{13}C NMR (125 MHz, $CDCl_3$, δ_C) 165.7 (C=O), 165.6 (C=O), 165.5(9) (C=O), 165.5 (C=O), 165.4 (C=O), 165.2 (C=O), 165.1(5) (C=O), 165.1 (C=O), 135.7 (Ar), 135.6 (Ar), 133.4 (Ar), 133.3 (Ar), 133.2(5) (Ar), 133.2 (Ar), 133.1 (Ar), 133.0(8) (Ar), 133.0 (Ar), 129.9 (Ar), 129.8(9) (Ar), 129.8(3) (Ar), 129.8 (Ar), 129.6 (Ar), 129.4 (Ar), 129.3 (Ar), 129.2(9) (Ar), 129.2 (Ar), 129.1 (Ar), 128.5 (Ar), 128.4 (Ar), 128.3 (Ar), 128.2(5) (Ar), 128.2 (Ar), 127.7 (Ar), 106.0 (C-1 × 2), 105.8 (C-1), 105.6 (C-1), 83.2, 82.1 (× 3), 81.9, 81.8, 81.6, 81.5, 77.4 (× 3), 77.3, 67.3 (octyl OCH_2), 66.0, 65.9, 65.8, 63.4, 51.4 (octyl CH_2N_3), 29.5 (octyl CH_2), 29.3 (octyl CH_2), 29.1 (octyl CH_2), 28.8 (octyl CH_2), 26.8 (*tert*-butyl $C(\mathit{C}H_3)_3$), 26.7 (octyl CH_2), 26.1 (octyl CH_2), 19.3 (*tert*-butyl $\mathit{C}(CH_3)_3$); HRMS (ESI) calcd for (M+Na) $C_{100}H_{99}N_3O_{25}SiNa$: 1792.6229, found 1792.6245.

8-Azidooctyl 2,3-Di-*O*-benzoyl-α-D-arabinofuranosyl-(1→5)-2,3-di-*O*-benzoyl-α-D-arabinofuranosyl-(1→5)-2,3-di-*O*-benzoyl-α-D-arabinofuranosyl-(1→5)-2,3-di-*O*-benzoyl-α-D-arabinofuranoside (7)

Prepared from compound **6** (1.12 g, 0.632 mmol) and HF-pyridine (0.6 ml) in pyridine-THF (1:4 v:v, 15 ml) as described for **4**, to afford **7** (0.921 g, 95%) as a syrup. R_f 0.21 (2:1, hexanes/EtOAc); $[\alpha]_D$ +11.2 (*c* 0.5, CH_2Cl_2); ^{1}H NMR (600 MHz, $CDCl_3$, δ_H) 8.06–8.02 (m, 10 H, Ar), 7.93–7.87 (m, 6 H, Ar), 7.60–7.38 (m, 18 H, Ar), 7.30–7.24 (m, 6 H, Ar), 5.67–5.62 (m, 6 H), 5.52 (d, 1 H, *J* = 1.3 Hz), 5.43 (d, 1 H, *J* = 0.9 Hz), 5.42–5.40 (m, 3 H, H-1 × 3), 5.22 (s, 1 H, H-1), 4.66–4.60 (m, 2 H), 4.47 (ddd, 1 H, *J* = 4.2, 4.2, 4.0 Hz), 4.44 (ddd, 1 H, *J* = 4.6, 4.6, 2.9 Hz), 4.23–4.15 (m, 3 H), 4.02–3.91 (m, 5 H), 3.76 (ddd, 1 H, *J* = 9.6, 6.7, 6.7 Hz, octyl OCH_2), 3.51 (ddd, 1 H, *J* = 9.6, 6.3, 6.3 Hz, octyl OCH_2), 3.22 (dd, 2 H, *J* = 7.0, 7.0 Hz, octyl CH_2N_3), 2.35 (br s, 1 H, OH), 1.68–1.54 (m, 4 H, octyl CH_2), 1.44–1.25 (m, 8 H, octyl CH_2); ^{13}C NMR (125 MHz, $CDCl_3$, δ_C) 166.0 (C=O), 165.7 (C=O × 2), 165.6 (C=O), 165.4 (C=O), 165.1 (C=O), 165.0(8) (C=O × 2), 133.5 (Ar), 133.4 (Ar), 133.3 (Ar), 133.2 (Ar), 133.1 (Ar), 129.9 (Ar), 129.8(5) (Ar), 129.8 (Ar), 129.7 (Ar), 129.4 (Ar), 129.2 (Ar), 129.1 (Ar), 129.0(4) (Ar), 129.0 (Ar), 128.5 (Ar), 128.4 (Ar), 128.3 (Ar), 128.2(5) (Ar), 105.9 (C-1 × 2), 105.8 (C-1), 105.6 (C-1), 83.7, 82.1, 82.0, 81.9, 81.8, 81.7, 81.6, 81.5, 77.7, 77.3 (× 2), 77.2(7), 67.3 (octyl OCH_2), 66.1, 66.0, 65.9, 62.3, 51.4 (octyl CH_2N_3), 29.5 (octyl CH_2), 29.3 (octyl CH_2), 29.1 (octyl CH_2), 28.8 (octyl CH_2), 26.6 (octyl CH_2), 26.1 (octyl CH_2); HRMS (ESI) calcd for (M+Na) $C_{84}H_{81}N_3O_{25}Na$: 1554.5051, found 1554.5047.

8-Azidooctyl 2,3,5-Tri-*O*-benzoyl-α-D-arabinofuranosyl-(1→5)-2,3-di-*O*-benzoyl-α-D-arabinofuranosyl-(1→5)-2,3-di-*O*-benzoyl-α-D-arabinofuranosyl-(1→5)-2,3-di-*O*-benzoyl-α-D-arabinofuranosyl-(1→5)-2,3-di-*O*-benzoyl-α-D-arabinofuranoside (9)

Prepared from thioglycoside **8**[2] (0.064 g, 0.113 mmol), alcohol **7** (0.144 g, 0.094 mmol), 4 Å molecular sieves (0.1 g), *N*-iodosuccinimide (0.032 g, 0.135 mmol) and silver triflate (0.004 g, 0.015 mmol) in CH_2Cl_2 (5 ml) as described for **3**, to afford **9** (0.163 g, 88%) as a syrup. R_f 0.36 (2:1, hexanes/EtOAc); $[\alpha]_D$ +19.9 (*c* 0.4, CH_2Cl_2); ^{1}H NMR (500 MHz, $CDCl_3$, δ_H) 8.08–7.99 (m, 14 H, Ar), 7.95–7.88 (m, 8 H, Ar), 7.60–7.36 (m, 23 H, Ar), 7.30–7.23 (m, 10 H, Ar), 5.70–5.64 (m, 8 H), 5.60 (d, 1 H, *J* = 4.7 Hz), 5.54 (d, 1 H, *J* = 1.0 Hz), 5.48 (s, 1 H), 5.44–5.42 (m, 3 H), 5.24 (s, 1 H), 4.84 (dd, 1 H, *J* = 11.8, 3.2 Hz), 4.76–4.72 (m, 1 H), 4.69–4.60 (m, 4 H), 4.47–4.44 (m, 1 H), 4.25–4.18 (m, 4 H), 4.01–3.92 (m, 4 H), 3.77 (ddd, 1 H, *J* = 9.6, 6.6, 6.6 Hz, octyl OCH_2), 3.52 (ddd, 1 H, *J* = 9.6, 6.3, 6.3 Hz, octyl OCH_2), 3.22 (dd, 2 H, *J* = 7.0, 7.0 Hz, octyl CH_2N_3), 1.68–1.53 (m, 4 H, octyl CH_2), 1.45–1.26 (m, 8 H, octyl CH_2); ^{13}C NMR (125 MHz, $CDCl_3$, δ_C) 166.2 (C=O), 165.7 (C=O × 2), 165.6(5) (C=O), 165.6 (C=O × 2), 165.5 (C=O), 165.2 (C=O), 165.1(3) (C=O), 165.1 (C=O × 2), 133.4 (Ar), 133.3 (Ar), 133.2 (Ar), 133.1(4) (Ar), 133.1 (Ar), 129.9 (Ar), 129.8 (Ar), 129.7 (Ar), 129.4 (Ar), 129.1 (Ar), 129.0 (Ar), 128.9 (Ar), 128.5 (Ar), 128.4 (Ar), 128.3 (Ar), 128.2 (Ar), 105.9(7) (C-1), 105.9 (C-1 × 2), 105.8 (C-1), 105.6 (C-1), 82.1, 82.0, 81.9(6), 81.9, 81.8, 81.6 (× 3), 81.2 (× 2), 77.8, 77.3 (× 2), 77.2 (× 2), 67.3 (octyl OCH_2), 66.0 (× 2), 65.9, 65.8, 63.7, 51.4 (octyl CH_2N_3), 29.5 (octyl CH_2), 29.3 (octyl CH_2), 29.1 (octyl CH_2), 28.8 (octyl CH_2), 26.6 (octyl CH_2), 26.1 (octyl CH_2); HRMS (ESI) calcd for (M+Na) $C_{110}H_{101}N_3O_{32}Na$: 1998.6260, found 1998.6268.

8-Azidooctyl α-D-arabinofuranosyl-(1→5)-α-D-arabinofuranosyl-(1→5)-α-D-arabinofuranosyl-(1→5)-α-D-arabinofuranosyl-(1→5)-α-D-arabinofuranosyl-(1→5)-α-D-arabinofuranoside (10)

To a solution of **9** (0.151 g, 0.015 mmol) in CH_2Cl_2 (1 ml) at room temperature was added a 0.1 M $NaOCH_3$ solution in CH_3OH (2 ml). The reaction mixture was stirred for 6 h, and was neutralized with the careful addition of Amberlyst-15 (H^+) cation exchange resin. The solution

was filtered and concentrated to give a syrup that was dissolved in distilled water (3 ml). The aqueous phase was washed with EtOAc (3 ml × 2), CH_2Cl_2 (3 ml) and the separated aqueous phase was lyophilized to give deprotected **10** (0.059 g, 94%) as a fluffy solid. $[\alpha]_D$ +132.5 (*c* 0.2, CH_3OH); ^{1}H NMR (500 MHz, D_2O, δ_H) 5.10–5.06 (m, 4 H, H-1 × 4), 5.00 (s, 1 H, H-1), 4.23–3.69 (m, 26 H, H-2 × 5, H-3 × 5, H-4 × 5, H-5 × 10, octyl OCH_2), 3.58–3.52 (m, 1 H, octyl OCH_2), 3.32 (dd, 2 H, *J* = 6.9, 6.9 Hz, octyl CH_2N_3), 1.65–1.58 (m, 4 H, octyl CH_2), 1.42–1.32 (m, 8 H, octyl CH_2); ^{13}C NMR (125 MHz, D_2O, δ_C) 108.5 (C-1), 108.4 (C-1 × 2), 108.3 (C-1), 108.2 (C-1), 84.8, 83.3 (× 2), 82.6, 81.9, 81.8 (× 2), 81.7(8), 81.7 (× 2), 77.7, 77.6 (× 2), 77.5, 77.4, 69.3 (octyl OCH_2), 67.7, 67.6 (× 2), 62.0 (× 2), 52.1 (octyl CH_2N_3), 29.6 (octyl CH_2), 29.4 (octyl CH_2), 29.3 (octyl CH_2), 29.0 (octyl CH_2), 26.9 (octyl CH_2), 26.1 (octyl CH_2); HRMS (ESI) calcd for (M+Na) $C_{33}H_{57}N_3O_{21}Na$: 854.3376, found 854.3382; FTIR 2096.9 cm^{-1} (N_3)

8-Aminooctyl α-D-arabinofuranosyl-(1→5)-α-D-arabinofuranosyl-(1→5)-α-D-arabinofuranosyl-(1→5)-α-D-arabinofuranosyl-(1→5)-α-D-arabinofuranoside (Ara5)

To a solution of **10** (0.0246 g, 0.029 mmol) in pyridine-H_2O (1:1 v:v, 2 ml) at room temperature was added 10% Pd-C (2 mg), and the reaction mixture was stirred vigorously under hydrogen (1 atm) for 14 h. The reaction mixture was diluted with methanol (5 ml) and filtered to remove the catalyst. The methanol solution was concentrated to give a syrup that was dissolved in distilled water (2 ml), filtered through Sep-Pak C18 Cartridge by flushing with water and then water/methanol (1:1 v:v). The fractions were combined, concentrated and lyophilized to give **Ara5** (0.0204 g, 86%) as a fluffy solid. ^{1}H NMR (500 MHz, D_2O, δ_H) 5.10–5.07 (m, 4 H, H-1 × 4), 5.02 (s, 1 H, H-1), 4.23–3.68 (m, 26 H, H-2 × 5, H-3 × 5, H-4 × 5, H-5 × 10, octyl OCH_2), 3.60–3.53 (m, 1 H, octyl OCH_2), 2.96 (dd, 2 H, *J* = 7.5, 7.5 Hz, octyl CH_2NH_2), 1.68–1.57 (m, 4 H, octyl CH_2), 1.41–1.30 (m, 8 H, octyl CH_2); ^{13}C NMR (125 MHz, D_2O, δ_C) 108.4 (C-1 × 3),

108.3 (C-1), 108.1 (C-1), 84.8, 83.2 (× 2), 82.6, 81.8, 81.7(3) (× 2), 81.7 (× 2), 77.7, 77.6 (× 2), 77.4 (× 2), 77.3, 69.5 (octyl OCH_2), 67.8 (× 2), 67.7, 62.1 (× 2), 40.5 (octyl CH_2NH_2), 29.4 (octyl CH_2), 29.0 (octyl CH_2), 28.9 (octyl CH_2), 27.9 (octyl CH_2), 26.4 (octyl CH_2), 25.9 (octyl CH_2); HRMS (ESI) calcd for (M+Na) $C_{33}H_{60}NO_{21}$: 806.3652, found 806.3652.

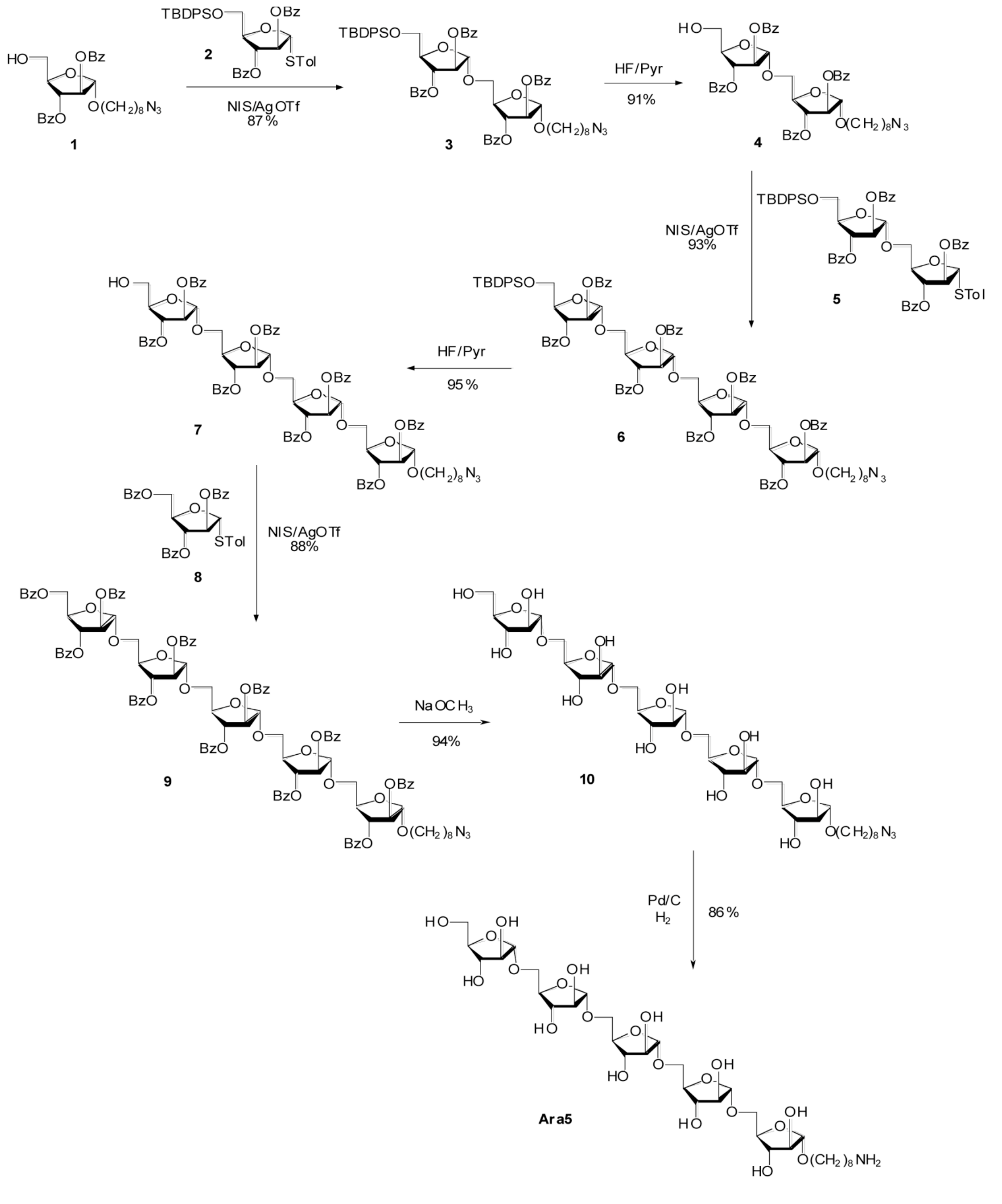

Figure S4

Reference:

(1) Joe, M.; Bai, Y.; Nacario, R. C.; Lowary, T. L. *J. Am. Soc. Chem.* **2007**, *127*, 9885-9901

(2) Callam, C. S.; Gadikota, R. R.; Lowary, T. L. *J. Org. Chem.* **2001**, *66*, 4549-4558
